# Problematic internet use (PIU): Associations with the impulsive-compulsive spectrum. An application of machine learning in psychiatry

**DOI:** 10.1016/j.jpsychires.2016.08.010

**Published:** 2016-12

**Authors:** Konstantinos Ioannidis, Samuel R. Chamberlain, Matthias S. Treder, Franz Kiraly, Eric W. Leppink, Sarah A. Redden, Dan J. Stein, Christine Lochner, Jon E. Grant

**Affiliations:** a Department of Psychiatry, University of Cambridge, UK; b Cambridge and Peterborough NHS Foundation Trust, Cambridge, UK; c Behavioural and Clinical Neuroscience Institute, University of Cambridge, UK; d University College London, Department of Statistical Science, London, UK; e Department of Psychiatry and Behavioral Neuroscience, University of Chicago, Chicago, IL, USA; f US/UCT MRC Unit on Anxiety & Stress Disorders, Department of Psychiatry, University of Stellenbosch, South Africa

**Keywords:** ADHD, Compulsivity, Impulsivity, Internet use, OCD, Machine learning

## Abstract

Problematic internet use is common, functionally impairing, and in need of further study. Its relationship with obsessive-compulsive and impulsive disorders is unclear. Our objective was to evaluate whether problematic internet use can be predicted from recognised forms of impulsive and compulsive traits and symptomatology. We recruited volunteers aged 18 and older using media advertisements at two sites (Chicago USA, and Stellenbosch, South Africa) to complete an extensive online survey. State-of-the-art out-of-sample evaluation of machine learning predictive models was used, which included Logistic Regression, Random Forests and Naïve Bayes. Problematic internet use was identified using the Internet Addiction Test (IAT). 2006 complete cases were analysed, of whom 181 (9.0%) had moderate/severe problematic internet use. Using Logistic Regression and Naïve Bayes we produced a classification prediction with a receiver operating characteristic area under the curve (ROC-AUC) of 0.83 (SD 0.03) whereas using a Random Forests algorithm the prediction ROC-AUC was 0.84 (SD 0.03) [all three models superior to baseline models p < 0.0001]. The models showed robust transfer between the study sites in all validation sets [p < 0.0001]. Prediction of problematic internet use was possible using specific measures of impulsivity and compulsivity in a population of volunteers. Moreover, this study offers proof-of-concept in support of using machine learning in psychiatry to demonstrate replicability of results across geographically and culturally distinct settings.

## 1 Introduction

The Internet has become an integral part of modern life, and has given rise to a wide range of problematic behaviors associated with its use (Cao et al., 2007). Some of those behaviors, like excessive online gaming, online buying and gambling, frequent email checking, prolific use of social media, and viewing pornography have been reported to cause significant impairment of everyday functioning of some individuals, to the extent that mental health professional help is sought or national health authorities are concerned (Choi, 2007, American Academy of Pediatrics, 2015)).

Epidemiological data have been gathered over the last two decades on problematic internet use (PIU) but the findings are mixed. Ko and colleagues (Ko et al., 2012) reported a prevalence of internet addiction that ranged from 1% to 36.7%. This huge variability in prevalence rates across studies could reflect differences in the assessment tools and different operational definitions of PIU behaviors. Other factors that might have contributed to this disparity of prevalence between studies are social, cultural, and demographic differences and inconsistencies of internet access. Could PIU represent a disorder in one country, but not a valid or relevant concept in another? In fact, internet activities are so widespread in 21st century youth, that there is anecdotal evidence that they have become an inescapable social norm (Wallace, 2014).

On an individual level, there have been strong suggestions that these PIU behaviors are linked with relationship difficulties, failure to thrive academically, and financial problems (Chang and Man Law, 2008, Király et al., 2015). Particularly young internet users have been reported to use online gaming compulsively, to the exclusion of other interests, and to experience significant impairment and distress as a result. Additionally, there has been anecdotal evidence of serious physical harm and death by cardiovascular collapse, the majority reported from East Asian countries, but also one case in the UK, in individuals who have engaged in ‘marathon’ internet sessions (more than 24 h of continuous activity) of mass multiplayer online gaming (Tam and Walter, 2013, Király et al., 2015).

The most recent literature suggests that some of these PIU behaviors are strongly linked with well identifiable mental health problems (Carli et al., 2013, Ho et al., 2014). A meta-analysis of eight studies comprising a total of 1641 patients with internet addiction and 11 210 controls found high correlations with mental disorders, including disorders of addiction *e.g.* alcohol use disorder (OR = 3.05) (Ko et al., 2008a, Yen et al., 2009a), affective disorders *e.g.* depression (OR = 2.77) (Ha et al., 2006, Ko et al., 2008b), anxiety disorders (OR = 2.70) *e.g.* generalized anxiety disorder (GAD), social anxiety disorder (SAD), obsessive-compulsive disorder (OCD), and attention-deficit hyperactivity disorder (ADHD, OR = 2.85) (Yoo et al., 2004, Yen et al., 2007, Yen et al., 2009b). The precise mapping of PIU onto other forms of psychopathology and other dimensions of behavior, like impulsivity and compulsivity, however, is relatively unexplored, and the associations derived from these studies are made under the not necessarily true assumption of a linear model, and have not been validated in terms of whether they really allow prediction of the presence of PIU. Further research is required as to how to fit the observed behavioral phenotypes of problematic internet use into a reliable and valid taxonomical system.

Machine learning (ML) is a subfield of computer science that involves the construction of algorithms that can learn and make predictions on data (Hastie et al., 2008). The main overall difference between traditional statistical models and machine learning techniques is that the latter enable prediction, usually on very few assumptions about the data (Breiman, 2001, Bishop, 2006). Traditional statistical models also enable prediction but usually based on specific assumptions about the data. In our study, we hypothesized that specific measures of impulsivity and compulsivity (self-rated ADHD symptoms (Kessler et al., 2005), along with questionnaire-based measures from the Barratt Impulsiveness Questionnaire (Patton et al., 1995), and the Padua Obsessive-Compulsive Inventory (Burns et al., 1996)) would allow construction of ML algorithms for the prediction of PIU in a population of volunteers. Furthermore, we hypothesized that the performance of the prediction models only including a baseline set of demographic and clinical variables would be enhanced significantly if impulsivity and compulsivity variables were added as predictor variables. If true, such results would be indicative of internet addiction having potentially clinically relevant relationships with these other types of symptomatology. Further reasons for using ML in this paper are described in the supplement (eMethods 1).

## 2 Methods

### 2.1 Setting and measures

The current study was conducted from January 2014–February 2015. Individuals aged 18 years and above were recruited at two sites: Chicago (USA) and Stellenbosch (South Africa) (mean age 30.1 [18–88]; 1316 males [65.6%]; 1447 Caucasian [72.1%]) using internet advertisements. The advertisements asked individuals to take part in an online survey about internet use. Participants completed the survey anonymously using Survey Monkey software. The survey was sent through Craigslist so only participants from the specific locales were targeted. The study was approved by the institutional review boards at each research site. Participants received no compensation for taking part but were enrolled in a random lottery whereby five prizes were available with each prize valued between $50 and $200 in USA and three prizes between ZAR250 and ZAR750 in South Africa.

The online survey contained questions about each individual's age, gender, race, and education background, along with various clinical measures. Clinical measures included the Internet Addiction Test (IAT) (Young, 1998), the Mini International Neuropsychiatric Interview (MINI) (Sheehan et al., 1998), the Padua Inventory (PI) (Burns et al., 1996), the Adult ADHD Self-Report Scale Symptom Checklist (ASRS-v1.1) (Kessler et al., 2005), and the Barratt Impulsiveness Scale (BIS-11) (Patton et al., 1995).

The IAT comprises 20 questions examining facets of PIU. Scores on the IAT range from 20 to 100 with 20–49 reflecting mild Internet use, 50–79 moderate Internet use, and 80–100 reflecting severe Internet use. The MINI is a brief structured interview for the major Axis I psychiatric disorders in the DSM-IV and ICD-10. For the purposes of the study, the MINI was adapted for self-administration and only included the OCD, SAD, and GAD modules. The latter was done to limit the length of the survey and ensure high completeness. The PI consists of 39 items assessing common obsessional and compulsive behavior. The ASRS-v1.1 is a self-report screening scale of adult ADHD. The BIS-11 is a self-report questionnaire used to determine levels of impulsiveness.

Only data of participants who completed the entirety of the online survey were included in the analyses. The original sample included 2566 individuals. 63 individuals were excluded for lacking IAT scores. Eighteen individuals were excluded for reporting a transgender gender. A further 474 individuals were excluded for missing important predictor variables *e.g.* ASRS, PI or BIS questionnaire scores. Five individuals were excluded for reporting age less than 18 years old. The final full set included 2006 individuals with complete scores in all variables. This final full set included 1316 individuals from the Stellenbosch site and 690 individuals from the Chicago site. All continuous predictors (i.e. age) were standardized to increase the interpretability of the model coefficients. The models classified individuals between non-problematic internet use (IAT score <50) and PIU (IAT score 50 and above). The same cut-off was used in the traditional statistics as well. All analyses were undertaken in R Studio version 3.1.2; ML was done using the caret package (Kuhn, 2015) (classification and regression training version “caret_6.0–47”). More details about the analysis process can be found in the supplement (eMethods 2).

### 2.2 Validation set-ups

In terms of validation set-ups, five different validation set-ups were chosen: (A) training and testing in the full data set, (B) training and testing in the Stellenbosch set, (C) training and testing in the Chicago set, (D) training in the Stellenbosch set and testing in the Chicago set, (E) training in the Chicago set and testing in the Stellenbosch set. The different site samples were used together as one sample in the full data set analysis (validation set-up A) and as separate sets during the within study site (validation set-ups B-C) and between study site analyses (validation set-ups D-E).

The process of training and testing the models was the same for all models. All analyses used cross-validation (Stone, 1974) with 50 replications and results were averaged. At each replication, the sample was partitioned in a training and a testing sub-sample which were complementary; in validation set-ups A, B and C this was done by randomly splitting the data set into a training (75%) and a testing (25%) partition. In validation set-ups D and E, training and testing sets were appointed by the way the set-up was defined. To avoid having identical training sets in each replication, only a random 90% of the available respective sample (Stellenbosch sample for validation set-up D and Chicago sample for validation set-up E) was used in each replication to train the model. Testing was done in the respective other sample (Chicago sample for validation set-up D and Stellenbosch sample for validation set-up E). A set seed was placed to allow replicability of results. The set seed was randomly selected by the researchers and was the same in all set ups and models. Every set was partitioned randomly into complementary training and testing sets using the caret package.

### 2.3 Error metrics

Receiver-operating characteristic area under the curve (ROC-AUC) and Precision-Recall area under the curve (PR-AUC) were used to examine the performance of the different models. This was considered the most suitable approach for a classification problem with unbalanced groups (Chawla, 2005). AUC is a useful and widely used metric in medical sciences, however, it lacks the ability to weight omission and commission errors and summarizes test performance in areas of the ROC space that are not always relevant for clinical practice (Lobo et al., 2008). Precision-recall curves (PR) to assess a models' performance are not widely used in medical sciences and lack the ability of taking into account of the true negative rate. However, PR curves well complement ROC curves in solving classification problems especially with highly skewed data sets (Davis and Goadrich, 2006). More metrics are reported in the online supplement, including accuracy, sensitivity, specificity, positive predictive value, negative predictive value, kappa and F-measure. Mean, standard deviation, and standard error of the mean was calculated for these metrics. Another output metric that was examined was variable importance (VI), which gives an indication of whether a variable is useful for an algorithm to make decisions. VI results were averaged reported in descending order.

### 2.4 Prediction methods

Three ML algorithms were used: Logistic Regression (LR), Random Forests (RF) (Breiman, 2001), and Naïve Bayes (NB) (Duda and Hart, 1973). A Random Forest is a combination of many binary decision trees. When the model receives new data, each decision tree produces a separate response and the overall output is determined by a majority vote. We used the default value of 500 trees. The number of variables considered at each node was a variable tuning parameter that was optimised by a tuning function. The Naive Bayes classifier applies Bayes rule to select the class that maximises the posterior probability of the class labels given the data. Probability distributions were based on kernel density estimates using the training data. No Laplace correction was applied.

Model construction and predictions were made using five different sets of variables: (a) a ‘baseline set’ of demographic variables, including age, sex, race, education plus social anxiety disorder and generalized anxiety disorder diagnoses, (b) a set that included all baseline variables plus impulsivity and compulsivity variables, (c) a set that included all baseline variables plus impulsivity variables only, (d) a set that included all baseline variables plus compulsivity variables only and (e) a set of demographic, impulsivity and compulsivity variables with randomized scores to establish the ‘chance’ baseline. An in-sample logistic regression was also fit to ascertain associations using a traditional approach.

## 3 Results

**Fig. 1:**
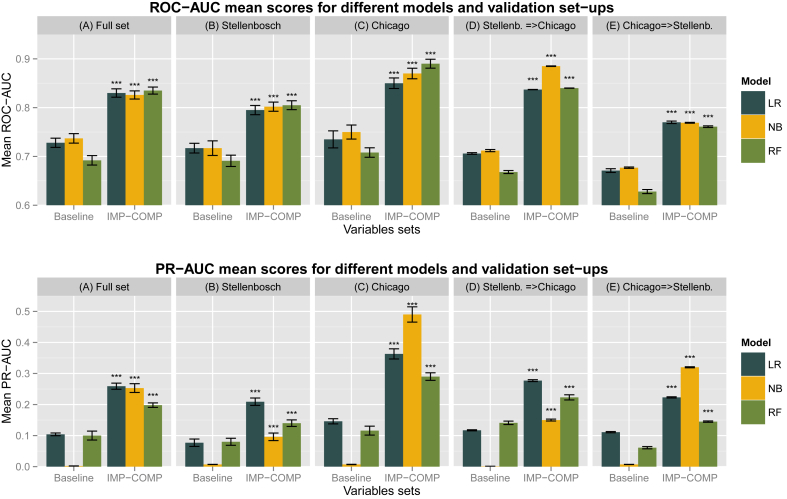
Summary figure of comparisons between models that included both impulsivity and compulsivity measures against baseline models in all validation set-ups. ROC-AUC: Receiver-operating characteristic Curve – Area Under the curve; PR-AUC: Precision-Recall curve – Area. under the curve; All p values are Wilcoxon signed rank test with continuity correction. All significant values support the alternative hypothesis that true location shift is not equal to zero and therefore models that included both impulsivity and compulsivity were superior to models with baseline variables only. IMP-COMP: Models that includes impulsivity and compulsivity variables as well as baseline variables. Baseline: Models that includes baseline variables only. Stellenb.=>Chicago: Models trained in the Stellenbosch set and tested on the Chicago set. Chicago=>Stellenb.: Models trained in the Chicago set and tested on the Stellenbosch set. Significance codes: ‘***’ <0.001 ‘**’ <0.01 ‘*’ <0.05 ‘.’ ≥0.05.

Complete data were available for 2006 subjects and all of those were included in the analyses. Demographic and clinical characteristics in the full sample are presented in Table 1. Demographic and clinical characteristics stratified by study site are presented in the supplement eTable 1 and eTable 2. Models that included impulsivity and compulsivity variables produced significantly higher ROC-AUC and PR-AUC from their respective baseline models in all five validation sets. A summary table of those results are presented in Fig. 1. Further head-to-head comparisons between models are presented in the supplement eTables 3–7. All model comparisons we performed using the Wilcoxon signed rank test. There are not any models that were tried and failed and not reported in the manuscript.

### 3.1 Full data set results

**Fig. 2:**
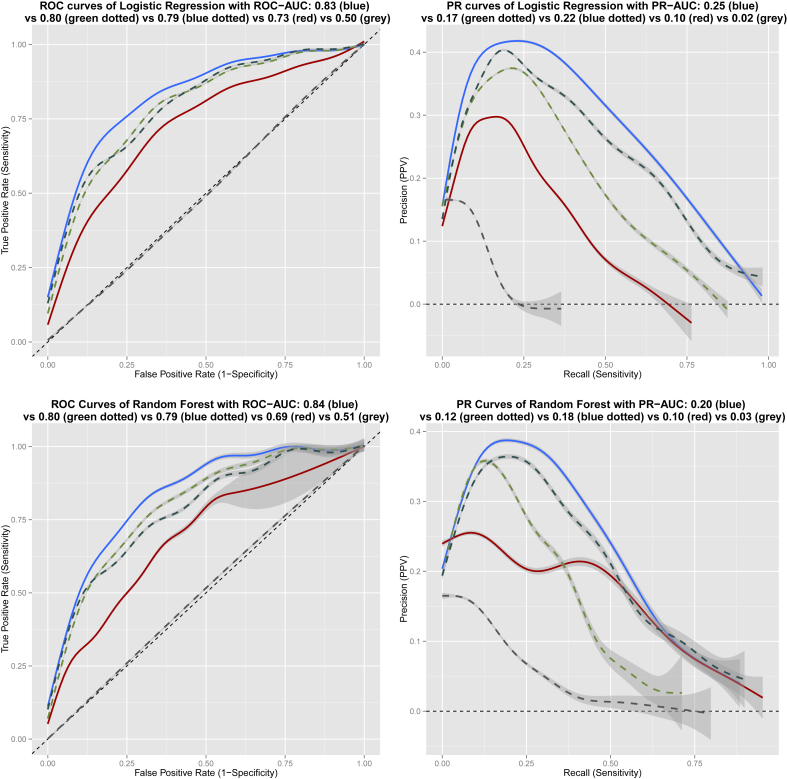
Receiver operating characteristic and Precision-Recall Curves for Logistic Regression and Random Forest Machine Learning prediction models trained and tested in the full data set. ‘Blue’ line: Prediction model curve using baseline plus impulsivity and compulsivity variables. ‘Green dotted’ line: Prediction model using baseline plus impulsivity variables. ‘Blue dotted’ line: Prediction model using baseline plus compulsivity variables. ‘Red’ line: Prediction model curve using baseline variables only. ‘Grey dotted’ line: Prediction model curve ‘at chance’ level with randomized variable scores.

In more detail, in the whole data set using the Logistic Regression algorithm we produced a classification prediction that could distinguish PIU from non-PIU with an ROC-AUC of 0.83 (SD 0.03) compared to baseline ROC-AUC 0.73 (SD 0.03) and PR-AUC 0.26 (SD 0.04) compared to baseline PR-AUC 0.10 (SD 0.02). Random Forests had an ROC-AUC of 0.84 (SD 0.03) compared to baseline ROC-AUC 0.69 (SD 0.03) and PR-AUC 0.20 (SD 0.03) compared to baseline PR-AUC 0.10 (SD 0.05). Naïve Bayes had an ROC-AUC of 0.83 (SD 0.03) compared to baseline ROC-AUC 0.74 (SD 0.04) and PR-AUC 0.25 (SD 0.05) compared to baseline PR-AUC 0.01 (SD 0.00). Variable importance rank averages from LR and RF are shown in Table 2. A graphic representation of the ROC and PR curves of those models is shown in Fig. 2. More metrics are presented in the supplement eTable 8 and eTable 9.

### 3.2 Within and between study sites results

We found that models including impulsivity and compulsivity variables outperformed their respective baseline models, both when exclusively trained and validated on one study site [*validation set-ups B and C*], but also when models were trained on data from one-study site and validated to independent data from the other study site and vice versa [*validation set-ups D and E*]. Results of within and between study sites analyses [*validation set-ups B-E*] including all metrics, ROC-AUC and PR-AUC scores and VI matrices are presented fully in the supplement eTables 10–17 and graphically presented in eFigures 2–5.

### 3.3 Chance-level results with randomized variable scores

All ‘chance level’ predictions conveyed ROC-AUCs close to 0.50 and PR-AUCs close to 0.0.

### 3.4 In-sample results using traditional statistical methods

In the complete data set PIU was associated with significantly elevated risk of OCD, ADHD, SAD, and GAD (all p < 0.001) [Table 1]. Using Logistic Regression, PIU was significantly strongly associated (in descending order of statistical significance) with older age (Z = 5.596), greater ADHD symptom severity (ASRS, Z = 5.303), non-Caucasian race (Z = 3.974), higher Padua ‘impulses to harm self/others’ (Z = 4.013), higher Padua ‘checking compulsions’ (Z = 3.407) (all p < 0.001), and higher Barratt Motor Impulsiveness (Z = 3.154, p = 0.0016) [Table 3].

### 3.5 Intermediate models comparisons

We introduced impulsivity and compulsivity sets of variables in a step-wise fashion to establish that both dimensions were important and able to improve predictions [eTable 3]. We compared models with impulsivity only or compulsivity only variables added to their baseline sets against the respective baseline sets. Impulsivity variables improved either ROC-AUC or PR-AUC significantly [p < 0.001] in 14 out of 15 comparisons. Compulsivity variables improved either ROC-AUC or PR-AUC significantly [p < 0.001] in all 15 comparisons [p < 0.001]. Combining impulsivity and compulsivity variables as predictors, compared to impulsivity or compulsivity alone, further improved either ROC-AUC or PR-AUC significantly [p < 0.001] in 29 out of 30 comparisons [eTable 4].

### 3.6 Between algorithms comparison

Overall, all three algorithms performed similarly in the full data set. LR and RF performed similarly in terms of ROC-AUC but LR outperformed RF in PR-AUC [eTable 5]. NB outperformed LR in terms of ROC-AUC in validation set-ups C and D only but performed variably in terms of PR-AUC [eTable 6]. NB outperformed RF in both between-sites cross-validation set-ups (D and E) but performed variably in terms of PR-AUC [eTable 7].

## 4 Discussion

### 4.1 Brief summary

This two-site original investigation showed that problematic internet use (PIU) can be predicted from a number of impulsivity and compulsivity variables, as well as baseline demographic and other clinical characteristics. Furthermore, the performance of the prediction models was significantly increased when sets of variables of impulsivity and compulsivity were added to the baseline variables of the prediction models. The inclusion of impulsivity and compulsivity together additively improved performance compared to each dimension used alone. Wilcoxon signed rank tests on ROC-AUC and PR-AUC scores to ascertain model comparisons established that all machine learning methods used (LR, NB and RF) performed similarly and were able to produce the above results in all validation set-ups. Moreover, the out-of-sample cross-validation between two study sites indicated that the predictive models were universal and robust, in that they permitted predictions across two geographically and culturally distinct settings. To our knowledge, this approach has not been utilized before in psychiatry, for any mental disorder. Our approach using ‘out-of-sample’ prediction means that we were able to estimate how well the models will perform in future, that is, it quantifies the predictive value of the statistical model. In contrast, this is not the case with traditional statistical methods, as commonly used in psychiatry to date, where significances decay in replication studies.

### 4.2 PIU and impulsivity

Previous studies have identified significant associations between PIU and high rates of impulsive disorders and symptomatology (Ko et al., 2009, Carli et al., 2013, Ho et al., 2014). Our study identified similar associations replicating previous results, but also ascertained that indicators of impulsivity, like ADHD and BIS-11 sub-scores (i.e. motor impulsivity, attentional impulsivity, non-planning impulsivity), are useful to make out-of-sample predictions of PIU, which adds to the validity to those associations and highlights the fact that impulsivity as a dimension, and not only as a categorical variable, is important for PIU. Particularly total ASRS score and motor impulsivity appear to be more important.

### 4.3 PIU and compulsivity

The importance of compulsivity has much less been identified in PIU (Bernardi and Pallanti, 2009, Pallanti, 2010), although specific types of problematic online behaviours have been identified to have compulsive components (King and Barak, 1999, Greenfield, 1999), (Wetterneck et al., 2012), (Weinstein et al., 2015a, Weinstein et al., 2015b). Our results showed that compulsivity variables are useful to make out-of-sample predictions of PIU, suggesting that compulsivity as a dimensional variable plays an important role in those behaviors and merits further investigation. Among PI variables, checking compulsions and obsessive impulses to harm self or others appeared to be more important.

### 4.4 PIU and demographic characteristics

Older age was linearly associated with higher rates of PIU in our sample, but stratification by study site showed that this association stemmed from the Stellenbosch sample only. Limited research has examined how adult populations with mental health problems behave online. In adult and late adult populations there is a considerable incidence and projected lifetime risk of psychiatric disorders commonly associated with PIU (Faraone et al., 2006, Cunningham-Williams et al., 2005, Kessler et al., 2007a, Kessler et al., 2007b), therefore it is important to explore how PIU and those disorders interact. Arguably, the relationship between age and PIU might be non-linear if assessed across the whole age span. Caucasian race was associated with lower rates of PIU at both study sites; this is a result that merits further investigation. Exploring how a similar analysis would hold in a setting with a majority of non-Caucasian populations is an idea worth considering; socio-cultural factors common to both study-sites used may be confounding this observed relationship.

In contrast to other PIU studies, we did not find any gender differences relating to PIU. However, our sample did not include adolescents. When problematic internet behaviors in adolescents were assessed in Korean youth, those were more prevalent in males (Ha and Hwang, 2014), nevertheless, similar structural brain changes have been identified in females with PIU (Altbäcker et al., 2015). In a recent study, about half of the individual differences in compulsive online behaviors were accounted for by genetic factors to an equal degree in both genders. It was furthermore noted that boys spend more time gaming while girls spend more time on social network sites and chatting (Vink et al., 2015). While it is plausible that gender differences are masked by selection of the study sample, ours and previous results imply that if a wider range of problematic online behaviors are assessed (and not only internet gaming), gender effects might weaken or disappear (Király et al., 2014). If gender differences in the presentation of PIU may be more pronounced in adolescents or young adults, those might stem from a neurobiological susceptibility of young males towards problematic online gaming or PIU in general.

### 4.5 Limitations

There are limitations to our study deriving from using the MINI; this is validated to be delivered from a trained person in a face-to-face interview whereas in our study it was delivered via an online tool. Given the strong links that are reported from previous studies between PIU and psychiatric diagnoses, it is likely that accurate or a wider variety of diagnostic data would improve the predictive accuracy of the models using diagnoses as predictors. Due to using Craigslist, we cannot exclude the possibility of a small number of non-local people having accessed the survey. However, participants were required to provide an address to enter the prize draw, thereby reducing the likelihood of non-local participants contributing to the survey. Our sample consisted of only 1% in the severe group (IAT ≥ 80) and we were unable to accurately assess classification metrics for predicting the severe group alone. A further limitation is that this study did not explore a wide variety of ML algorithms. For the purposes of this study we focused only on three ML methods that all confirmed our hypotheses and demonstrated the proof-of-concept.

### 4.6 Classification controversy of problematic internet use

There is still a debate as to how to fit the observed behavioral phenotypes of problematic internet use into a reliable and valid taxonomical system. Despite an accumulation of empirical data and analyses on internet addiction behaviors, any clear theoretical conclusions are currently lacking. Since the introduction of the term “Internet Addiction disorder” in the mid-nineties many attempts have been made to revisit the proposed diagnostic criteria, refining the assessment tools (Koh, 2007, Lortie and Guitton, 2013) and formalize the concept in the new classification systems (Block, 2008). Internet gaming has been shown to excessively boost the brain reward systems, while deficits of the dopaminergic system have been identified in internet gaming addiction. Recent imaging data show that the reward, addiction, craving and emotion circuits in the brain are increasingly activated during gaming activities. Therefore, categorizing problematic internet use as an addiction disorder, seems to hold the strongest biological footing and has dominated the literature on the field so far (Kuss and Griffiths, 2012). At the same time, there is a wide range of internet activities that have been observed to have compulsive elements and share commonalities with impulse control disorders; this has raised the question whether problematic internet use should better be classified as an impulse control disorder or within the impulsive-compulsive or obsessive-compulsive spectrum. Modern psychiatric classification systems are undergoing scrutiny and well-deserved critique for their epistemological failings, lack of biological grounding and weak validity (Aragona, 2009). When exploring new concepts like PIU, there is a need for different approaches in psychiatry, that would provide stronger links between behavioral phenotypes observed and brain biology (Cuthbert and Insel, 2013, Cuthbert, 2014), approaches that would allow dimensional constructs to enrich the descriptive frameworks and strengthen the validity and generalizability of the results produced (Hyman, 2010, Nesse and Stein, 2012).

### 4.7 Is PIU a meaningful diagnostic entity?

Although this study does not explore whether PIU shares elements with addictions, it adds to the clinical description of problematic internet behaviors, thus contributing to achieving a valid classification. Furthermore, it strengthens the argument that PIU, if it is to be regarded as a disorder in its own right, should likely be categorized within the impulsive-compulsive spectrum. Such categorization might open several new areas of investigation. PIU could be considered as a newly identified area of symptomatology for the disorders of that spectrum i.e. impulsive online buying in the context of ADHD or compulsive use of social media in the context of OCD, which would respond to well-established treatments for these disorders, or it might worth be considered as a separate commonly co-morbid disorder, requiring PIU-specific treatments. In terms of prevalence rates, individuals suffering from disorders of the impulsive-compulsive spectrum might be at more risk of developing PIU or more severe forms of it. Treating psychiatric co-morbidities as early as possible has been suggested to prevent the development of pathological use of the internet (Ko et al., 2009). In terms of prevention, early identification of PIU may facilitate the diagnosis of impulsive-compulsive disorders and other related common health problems, and enable timely management of a wide range of mental health difficulties. Finally, it will be important to develop better assessment tools for PIU and evidence-based management strategies, which are currently lacking (Weinstein and Lejoyeux, 2010). There is only preliminary evidence for pharmacological treatments of PIU which are mainly conceived and focused on treating a co-morbid disorder, for example treating PIU symptoms by treating co-morbid ADHD with methylphenidate. Psychological treatments including individual or group Cognitive behavioral therapy, family based interventions, and motivational interviewing have been suggested as a possible treatments for PIU symptoms (Spada, 2014).

### 4.8 Broader applications of machine learning for psychiatry in general

In terms of the methodology used, this study demonstrates a proof-of-concept for the use of machine learning approaches with behavioral data in psychiatry, with special consideration to the use of between-study-sites cross-validation. Such approaches enable multi-site studies to explore how robustly the results transfer between distinct settings, which is a vital step in establishing the ‘validity’ of a given mental disorder.

## Author contributions

KI designed the idea for the manuscript, analysed the data, wrote the majority of the manuscript and supplementary material and coordinated the co-authors' contributions. SRC, MT and FK participated in the conception and review of the statistical analysis. SRC, EL, SR, DJS, CL and JEG designed and coordinated the study and collected and managed the data. All authors read and approved the final manuscript and contributed to the drafting and revising of the paper as well as to interpreting the results.

## Role of the funding source

This research received internal departmental funds of the Department of Psychiatry at the University of Chicago. Authors received no funding for the preparation of this manuscript. The funding source played no role in the design, data analysis, or writing of the study.

## Conflict of interests

Dr. Grant has received research grants from NIDA, the National Center for Responsible Gaming, and Roche and Forest Pharmaceuticals. Dr. Grant receives compensation from Springer as the editor-in-chief of the Journal of Gambling Studies and has received royalties from McGraw Hill, Oxford University Press, Norton, and the APPI. Dr. Chamberlain consults for Cambridge Cognition and his involvement in this research was supported by a grant from the Academy of Medical Sciences (AMS, UK) and by an Intermediate Clinical Fellowship from the Wellcome Trust (UK; 110049/Z/15/Z). Dan Stein and Christine Lochner are funded by Medical Research Council of South Africa. The other authors report no financial relationships with commercial interest.

## Figures and Tables

**Table 1:** Demographic and clinical characteristics in the full sample (n = 2006, controls = 1825, cases = 181).

Variable	IAT score <50^a^	IAT score ≥ 50^b^	p-value	Corrected p-value (*177)^c^	Effect size^d^
IAT scores (SD)	30.6 (7.3)	59.9 (9.8)	<0.0001 v	<0.0001 v	0.57
Age, years (SD)	29.8 (13.3)	33.2 (14.3)	<0.0010 v	0.1685 v	
Gender, male, n (%)	1199 (65.6)	117 (64.6)	0.8386	>0.99	
Race, Caucasian, n (%)	1345 (73.6)	102 (56.3)	<0.0001	0.0002	0.11
Education, n (%) Below high school	12 (0.6)	1 (0.6)			
High school graduate	198 (10.8)	26 (14.3)			
Some college	444 (24.3)	68 (37.5)	0.0001	0.0253	0.10
College graduate	740 (40.5)	63 (34.8)			
Beyond College	431 (23.6)	23 (12.7)			
GAD, n (%)	322 (17.6)	78 (43.1)	<0.0001	<0.0001	0.18
Social Anxiety Disorder, n (%)	209 (11.4)	58 (32.0)	<0.0001	<0.0001	0.17
ADHD, n (%)	753 (41.2)	131 (72.3)	<0.0001	<0.0001	0.18
OCD, n (%)	159 (8.7)	50 (27.6)	<0.0001	<0.0001	0.17

a Internet addiction test (IAT) score <50 (Controls n = 1825).

b IAT score ≥ 50 (problematic internet use n = 181); All scores are mean (SD) unless otherwise noted. Statistic: chi-square except where indicated with ‘v’ ANOVAs for. Numbers in parentheses are percentages of each element in the respective groups. GAD: Generalized Anxiety Disorder; ADHD: Attention-Deficit Hyperactivity Disorder; OCD: Obsessive-Compulsive Disorder.

c Bonferroni correction applied.

d Effect sizes are eta squared for ANOVA and phi for chi square tests.

**Table 2:** Overview of variable importance results of Logistic Regression and Random Forest models listed by averaged variable importance ranks from all sets – only first 15 items displayed.

Variable	VI rank average
Race (non-Caucasian)	2.6
Age (older)	3.2
Impulses to harm self or others (PADUA)	3.8
Checking compulsion (PADUA)	4
Motor impulsivity (BIS)	5
ASRS	7.6
ADHD diagnosis	8
PADUA dressing grooming Compulsions (PADUA)	8.8
GAD diagnosis	9.6
Attention impulsivity (BIS)	10.6
PADUA contamination obsessions and washing compulsions (PADUA)	10.8
Social Anxiety Diagnosis	11.8
Thoughts of harm to self or others (PADUA)	12.2
Non-planning impulsivity (BIS)	12.6
OCD diagnosis	13
…	…

ADHD – Attention Deficit Hyperactivity Disorder; ASRS – Adult ADHD Self-Report Scale (ASRS-v1.1); BIS – Barratt Impulsiveness Scale 11; GAD – Generalized Anxiety disorder; OCD – Obsessive-Compulsive disorder; PADUA – Padua Inventory-Revised; VI – Variable importance.

**Table 3:** Logistic Regression model in the full data set (in-sample), with problematic internet use category (moderate and severely problematic versus controls) as dependent variable.

Variable	Estimate ± Std. Error	z value	Pr(>|z|)
Age	0.51 ± 0.09	5.59	<0.0001
Gender	−0.12 ± 0.19	−0.63	0.5304
Race	0.74 ± 0.19	3.97	<0.0001
Education	0.71 ± 1.24	0.57	0.5681
	0.69 ± 1.23	0.56	0.5773
	0.43 ± 1.23	0.35	0.7293
	0.02 ± 1.25	0.02	0.9845
ASRS	0.52 ± 0.01	5.31	<0.0001
Attention impulsivity (BIS)	0.15 ± 0.12	1.22	0.2213
Motor impulsivity (BIS)	0.37 ± 0.12	3.15	0.0016
Non-planning impulsivity (BIS)	0.05 ± 0.10	0.53	0.5981
Checking compulsion (PADUA)	0.41 ± 0.12	3.41	0.0007
PADUA contamination obsessions and washing compulsions (PADUA)	−0.02 ± 0.11	−0.17	0.8681
PADUA dressing grooming compulsions (PADUA)	0.16 ± 0.10	1.69	0.0904
Impulses to harm self or others (PADUA)	0.32 ± 0.08	4.01	<0.0001
Thoughts of harm to self or others (PADUA)	0.11 ± 0.11	0.97	0.3305

ASRS – Adult ADHD Self-Report Scale (ASRS-v1.1) Symptom Checklist; BIS – Barratt Impulsiveness Scale 11; PADUA – Padua Inventory-Revised.

## References

[bib1] Altbäcker A., Plózer E., Darnai G., Perlaki G., Horváth R., Orsi G. (2015). Problematic internet use is associated with structural alterations in the brain reward system in females. Brain Imaging Behav..

[bib2] American Academy of Pediatrics (2015). Media and Children. https://www.aap.org/en-us/advocacy-and-policy/aap-health-initiatives/Pages/Media-and-Children.aspx.

[bib3] Aragona M. (2009). The role of comorbidity in the crisis of the current psychiatric classification system. Philos., Psychiatr. Psychol..

[bib4] Bernardi S., Pallanti S. (2009). Internet addiction: a descriptive clinical study focusing on comorbidities and dissociative symptoms. Compr. Psychiatry.

[bib5] Bishop Christopher (2006). Pattern Recognition and Machine Learning.

[bib6] Block J.J. (2008). Issues for DSM-V: internet addiction. Am. J. Psychiatry.

[bib7] Breiman L. (2001). Random Forests. Mach. Learn..

[bib8] Burns G.L., Keortge S.G., Formea G.M., Sternberger L.G. (1996). Revision of the Padua inventory of obsessive compulsive disorder symptoms: distinctions between worry, obsessions, and compulsions. Behav. Res. Ther..

[bib9] Cao F., Su L., Liu T., Gao X. (2007). The relationship between impulsivity and internet addiction in a sample of chinese adolescents. Eur. Psychiatry J. Assoc. Eur. Psychiatr..

[bib10] Carli V., Durkee T., Wasserman D., Hadlaczky G., Despalins R., Kramarz E. (2013). The association between pathological internet use and comorbid psychopathology: a systematic review. Psychopathology.

[bib11] Chang M.K., Man Law S.P. (2008). Factor structure for Young's internet addiction test: a confirmatory study. Comput. Hum. Behav..

[bib12] Chawla N.V., Maimon Oded, Rokach Lior (2005). Data mining for imbalaned datasets: an Overview. Data Mining and Knowledge Discovery Handbook.

[bib13] Choi Y.H. (2007). Advancement of IT and seriousness of youth internet addiction. International Symposium on the Counseling and Treatment of Youth Internet Addiction.

[bib14] Cunningham-Williams R.M., Grucza R.A., Cottler L.B., Womack S.B., Books S.J., Przybeck T.R. (2005). “Prevalence and predictors of pathological gambling: results from the st. Louis personality, health and lifestyle (SLPHL) study. J. Psychiatr. Res..

[bib15] Cuthbert B.N. (2014). The RDoC framework: facilitating transition from ICD/DSM to dimensional approaches that integrate neuroscience and psychopathology. World Psychiatry Off. J. World Psychiatr. Assoc. (WPA).

[bib16] Cuthbert B.N., Insel T.R. (2013). Toward the future of psychiatric diagnosis: the seven pillars of RDoC. BMC Med..

[bib17] Davis J., Goadrich M. (2006). The relationship between precision-recall and ROC curves. Proceedings of the 23rd International Conference on Machine Learning.

[bib18] Duda R.O., Hart P.E. (1973). Pattern Classification and Scene Analysis.

[bib19] Faraone S.V., Biederman J., Mick E. (2006). The age-dependent decline of attention deficit hyperactivity disorder: a meta-analysis of follow-up studies. Psychol. Med..

[bib20] Greenfield D.N. (1999). Psychological characteristics of compulsive internet use: a preliminary analysis. Cyberpsychol. Behav. Impact Internet, Multimedia Virtual Real. Behav. Soc..

[bib21] Ha J.H., Yoo H.J., Cho I.H., Chin B., Shin D., Kim J.H. (2006). Psychiatric comorbidity assessed in Korean children and adolescents who screen positive for internet addiction. J. Clin. Psychiatry.

[bib22] Hastie T., Tibshirani R., Friedman J. (2008). The Elements of Statistical Learning Data Mining, Inference, and Prediction.

[bib23] Ha Y.-M., Hwang W.J. (2014). Gender differences in internet addiction associated with psychological health indicators among adolescents using a national web-based survey. Int. J. Ment. Health Addict..

[bib24] Ho R.C., Zhang M.W.B., Tsang T.Y., Toh A.H., Pan F., Lu Y. (2014). The association between internet addiction and psychiatric Co-Morbidity: a meta-analysis. BMC Psychiatry.

[bib25] Hyman S.E. (2010). The diagnosis of mental disorders: the problem of reification. Annu. Rev. Clin. Psychol..

[bib26] Kessler R.C., Adler L., Ames M., Demler O., Faraone S., Hiripi E. (2005). The world health organization adult ADHD self-report scale (ASRS): a short screening scale for use in the general population. Psychol. Med..

[bib27] Kessler R.C., Amminger G.P., Aguilar-Gaxiola S., Alonso J., Lee S., Ustun B.T. (2007). Age of onset of mental disorders: a review of recent literature. Curr. Opin. Psychiatry.

[bib28] Kessler R.C., Angermeyer M., Anthony J.C., DE Graaf R., Demyttenaere K., Gasquet I. (2007). Lifetime prevalence and age-of-onset distributions of mental disorders in the world health Organization's world mental health survey initiative. World Psychiatry Off. J. World Psychiatr. Assoc. (WPA).

[bib29] King S.A., Barak A. (1999). Compulsive internet gambling: a new form of an old clinical pathology. Cyberpsychol. Behav. Impact Internet Multimedia Virtual Real. Behav. Soc..

[bib30] Király O., Griffiths M.D., Demetrovics Z. (2015). Internet gaming disorder and the DSM-5: conceptualization, debates, and controversies. Curr. Addict. Rep..

[bib31] Király O., Griffiths M.D., Urbán R., Farkas J., Kökönyei G., Elekes Z., Tamás D., Demetrovics Z. (2014). Problematic internet use and problematic online gaming are not the same: findings from a large nationally representative adolescent sample. Cyberpsychol. Behav. Soc. Netw..

[bib32] Ko C.-H., Yen J.-Y., Yen C.-F., Chen C.-S., Weng C.-C., Chen C.-C. (2008). The association between internet addiction and problematic alcohol use in adolescents: the problem behavior model. Cyberpsychol. Behav. Impact Internet, Multimedia Virtual Real. Behav. Soc..

[bib33] Ko C.-H., Yen J.-Y., Chen C.-S., Chen C.-C., Yen C.-F. (2008). Psychiatric comorbidity of internet addiction in college students: an interview study. CNS Spectr..

[bib34] Ko C.-H., Yen J.-Y., Chen C.-S., Yeh Y.-C., Yen C.-F. (2009). Predictive values of psychiatric symptoms for internet addiction in adolescents: a 2-Year prospective study. Arch. Pediatr. Adolesc. Med..

[bib35] Ko C.-H., Yen J.-Y., Yen C.-F., Chen C.-S., Chen C.-C. (2012). The association between internet addiction and psychiatric disorder: a review of the literature. Eur. Psychiatr. J. Assoc. Eur. Psychiatr..

[bib36] Koh Y.S. (2007). Development and application of K-scale as diagnostic scale for Korean internet addiction. International Symposium on the Counseling and Treatment of Youth Internet Addiction.

[bib37] Kuhn M. (2015). Caret: Classification and Regression Training (Version 6.0–52). https://cran.r-project.org/web/packages/caret/index.html.

[bib38] Kuss D.J., Griffiths M.D. (2012). Internet and gaming addiction: a systematic literature review of neuroimaging studies. Brain Sci..

[bib39] Lobo J.M., Jiménez-Valverde A., Real R. (2008). AUC: a misleading measure of the performance of predictive distribution models. Glob. Ecol. Biogeogr..

[bib40] Lortie C.L., Guitton M.J. (2013). Internet addiction assessment tools: dimensional structure and methodological status. Addict. (Abingdon, England).

[bib41] Nesse R.M., Stein D.J. (2012). Towards a genuinely medical model for psychiatric nosology. BMC Med..

[bib42] Pallanti S. (2010). Problematic internet use: is it more compulsory than rewarding or mood driven?. World Psychiatry Off. J. World Psychiatr. Assoc. (WPA).

[bib43] Patton J.H., Stanford M.S., Barratt E.S. (1995). Factor structure of the Barratt impulsiveness scale. J. Clin. Psychol..

[bib44] Sheehan D.V., Lecrubier Y., Sheehan K.H., Amorim P., Janavs J., Weiller E. (1998). The mini-international neuropsychiatric interview (M.I.N.I.): the development and validation of a structured diagnostic psychiatric interview for DSM-IV and ICD-10. J. Clin. Psychiatry.

[bib45] Spada M.M. (2014). An Overview of problematic internet use. Addict. Behav..

[bib46] Stone M. (1974). Cross-validatory choice and assessment of statistical predictions. J. R. Stat. Soc. Ser. B Methodol..

[bib47] Tam P., Walter G. (2013 Dec). Problematic internet use in childhood and youth: evolution of a 21st century affliction. Australas. Psychiatry.

[bib48] Vink J.M., van Beijsterveldt T.C.E.M., Huppertz C., Bartels M., Boomsma D.I. (2015 Mar). Heritability of compulsive internet use in adolescents. Addict. Biol..

[bib49] Wallace P. (2014). Internet addiction disorder and youth: there are growing concerns about compulsive online activity and that this could impede students' performance and social lives. EMBO Rep..

[bib50] Weinstein A., Mezig H., Mizrachi S., Lejoyeux M. (2015). A study investigating the association between compulsive buying with measures of anxiety and obsessive-compulsive behavior among internet shoppers. Compr. Psychiatry.

[bib51] Weinstein A.M., Lejoyeux M. (2010). Internet addiction or excessive internet use. Am. J. Drug Alcohol Abuse.

[bib52] Weinstein A.M., Zolek R., Babkin A., Cohen K., Lejoyeux M. (2015). Factors predicting cybersex use and difficulties in forming intimate relationships among male and female users of cybersex. Front. Psychiatry.

[bib53] Wetterneck CT, Burgess AJ, Short MB, Smith AH, Cervantes ME. 2012. The role of sexual compulsivity, impulsivity, and experiential avoidance in internet pornography use 62(1): 3–18.

[bib54] Yen J.-Y., Ko C.-H., Yen C.-F., Chen C.-S., Chen C.-C. (2009). The association between harmful alcohol use and internet addiction among college students: comparison of personality. Psychiatry Clin. Neurosci..

[bib55] Yen J.-Y., Ko C.-H., Yen C.-F., Wu H.-Y., Yang M.-J. (2007). The comorbid psychiatric symptoms of internet addiction: attention deficit and hyperactivity disorder (ADHD), Depression, Social Phobia, and Hostility. J. Adolesc. Health Off. Publ. Soc. Adolesc. Med..

[bib56] Yen J.-Y., Yen C.-F., Chen C.-S., Tang T.-C., Ko C.-H. (2009). The association between adult ADHD symptoms and internet addiction among college students: the gender difference. Cyberpsychol. Behav. Impact Internet, Multimedia Virtual Real. Behav. Soc..

[bib57] Yoo H.J., Cho S.C., Ha J., Yune S.K., Kim S.J., Hwang J. (2004). Attention deficit hyperactivity symptoms and internet addiction. Psychiatry Clin. Neurosci..

[bib58] Young K.S. (1998). Internet addiction: the emergence of a new clinical disorder. CyberPsychol. Behav..

