# Supplementary material for: Problematic internet use (PIU): Associations with the impulsive-compulsive spectrum. An application of machine learning in psychiatry
Source: J Psychiatr Res. 2016 Dec;83:94–102. doi: 10.1016/j.jpsychires.2016.08.010 (PMC5119576; doi:10.1016/j.jpsychires.2016.08.010)
Supplement: Supplementary file 1 [file mmc1.docx]

Contents

[eMethods 1– Why use Machine learning? 3](#_Toc444463561)

[eMethods 2 – Data analysis details 4](#_Toc444463562)

[eMethods 3 – Why report extra metrics? 4](#_Toc444463563)

[eTable 1 – Demographic and clinical characteristics of the Stellenbosch sample 6](#_Toc444463564)

[eTable 2 – Demographic and clinical characteristics of the Chicago sample 7](#_Toc444463565)

[eTable 3 - Summary of comparisons between models that include impulsivity or compulsivity measures against models with baseline variables only 8](#_Toc444463566)

[eTable 4 - Summary of comparisons between models that include both impulsivity and compulsivity measures against models with impulsivity or compulsivity only 9](#_Toc444463567)

[eTable 5 – Head-to-head comparisons between Logistic Regression and Random Forests that include both impulsivity and compulsivity measures 10](#_Toc444463568)

[eTable 6 – Head-to-head comparisons between Logistic Regression and Naïve Bayes models that include both impulsivity and compulsivity measures 11](#_Toc444463569)

[eTable 7 – Head-to-head comparisons between Random Forests and Naïve Bayes models that include both impulsivity and compulsivity measures 12](#_Toc444463570)

[eTable 8: Validation set-up (A) Full data basic metrics for all models 13](#_Toc444463571)

[eTable 9: Validation set-up (A) Full data Variable Importance matrices from prediction using baseline plus impulsivity-compulsivity variables of Logistic Regression and Random Forests 14](#_Toc444463572)

[eFigure 1: Validation set-up (A) Full data Receiver Operating Characteristic and Precision-Recall Curves for all models 15](#_Toc444463573)

[eTable 10: Validation set-up (B) Stellenbosch set basic metrics for all models 16](#_Toc444463574)

[eTable 11: Validation set-up (B) Stellenbosch set Variable Importance matrices from prediction using baseline plus impulsivity-compulsivity variables of Logistic Regression and Random Forests 17](#_Toc444463575)

[eFigure 2: Validation set-up (B) Stellenbosch set Receiver Operating Characteristic and Precision-Recall Curves for all models 18](#_Toc444463576)

[eTable 12: Validation set-up (C) Chicago set basic metrics for all models 19](#_Toc444463577)

[eTable 13: Validation set-up (C) Chicago set Variable Importance matrices from prediction using baseline plus impulsivity-compulsivity variables of Logistic Regression and Random Forests 20](#_Toc444463578)

[eFigure 3: Validation set-up (C) Chicago set Receiver Operating Characteristic and Precision-Recall Curves for all models 21](#_Toc444463579)

[eTable 14: Validation set-up (D) trained on the Stellenbosch set and tested on the Chicago set basic metrics for all models 22](#_Toc444463580)

[eTable 15: Validation set-up (D) trained on the Stellenbosch set and tested on the Chicago set Variable Importance matrices from prediction using baseline plus impulsivity-compulsivity variables of Logistic Regression and Random Forests 23](#_Toc444463581)

[eFigure 4: Validation set-up (D) trained on the Stellenbosch set and tested on the Chicago set Receiver Operating Characteristic and Precision-Recall Curves for all models 24](#_Toc444463582)

[eTable 16: Validation set-up (E) trained on the Chicago set and tested on the Stellenbosch set basic metrics for all models 25](#_Toc444463583)

[eTable 17: Validation set-up (E) trained on the Chicago set and tested on the Stellenbosch set Variable Importance matrices from prediction using baseline plus impulsivity-compulsivity variables of Logistic Regression and Random Forests 26](#_Toc444463584)

[eFigure 5: Validation set-up (E) trained on the Chicago set and tested on the Stellenbosch set Receiver Operating Characteristic and Precision-Recall Curves for all models 27](#_Toc444463585)

[R code for the analysis (example) 28](#_Toc444463586)

[Appendix References 30](#_Toc444463587)

# eMethods 1– Why use Machine learning?

The main overall difference between traditional statistical models and machine learning techniques is that the latter enable prediction, usually on very few assumptions about the data (Breiman 2001a) (Bishop 2006). Traditional statistical models also enable prediction but usually based on specific assumptions about the data. Medical science is massively focused on data models and this has received criticism from some quarters of having led to irrelevant theory and questionable scientific conclusions (Breiman 2001a). Recursive partitioning methods like classification and regression trees, bagging and random forests are gaining popularity and becoming widely used tools for nonparametric regression and classification in many fields over the last decade or more(Bureau et al. 2005). Still, clinicians are largely unfamiliar with those methods.

Cross-validation (Stone 1974), is a common process in machine learning during which data is partitioned in multiple complementary subsets; one part of the data is held out and the other parts are used for estimating the model parameters (model training). The hold-out set is then used to estimate the predictive accuracy of the model (model validation). In contrast, in traditional statistical modelling, the full dataset is used for both model training and model validation. This procedure is usually repeated multiple times and results averaged. This process enables a more realistic estimation of a future out-of-sample prediction error and limits problems like over-fitting. What is important with this process is that validating out-of-sample prevents the common phenomenon that significances decay in replication studies when the original model is fitted in-sample. In our study, out-of-sample validation is also done using two different study sites and this represents an out-of-sample validation that strengthens the conclusions produced. There seems to be scope for machine learning methods that use cross-validation for multi-site studies to support the generalizability of any findings, and validity of any underlying concepts.


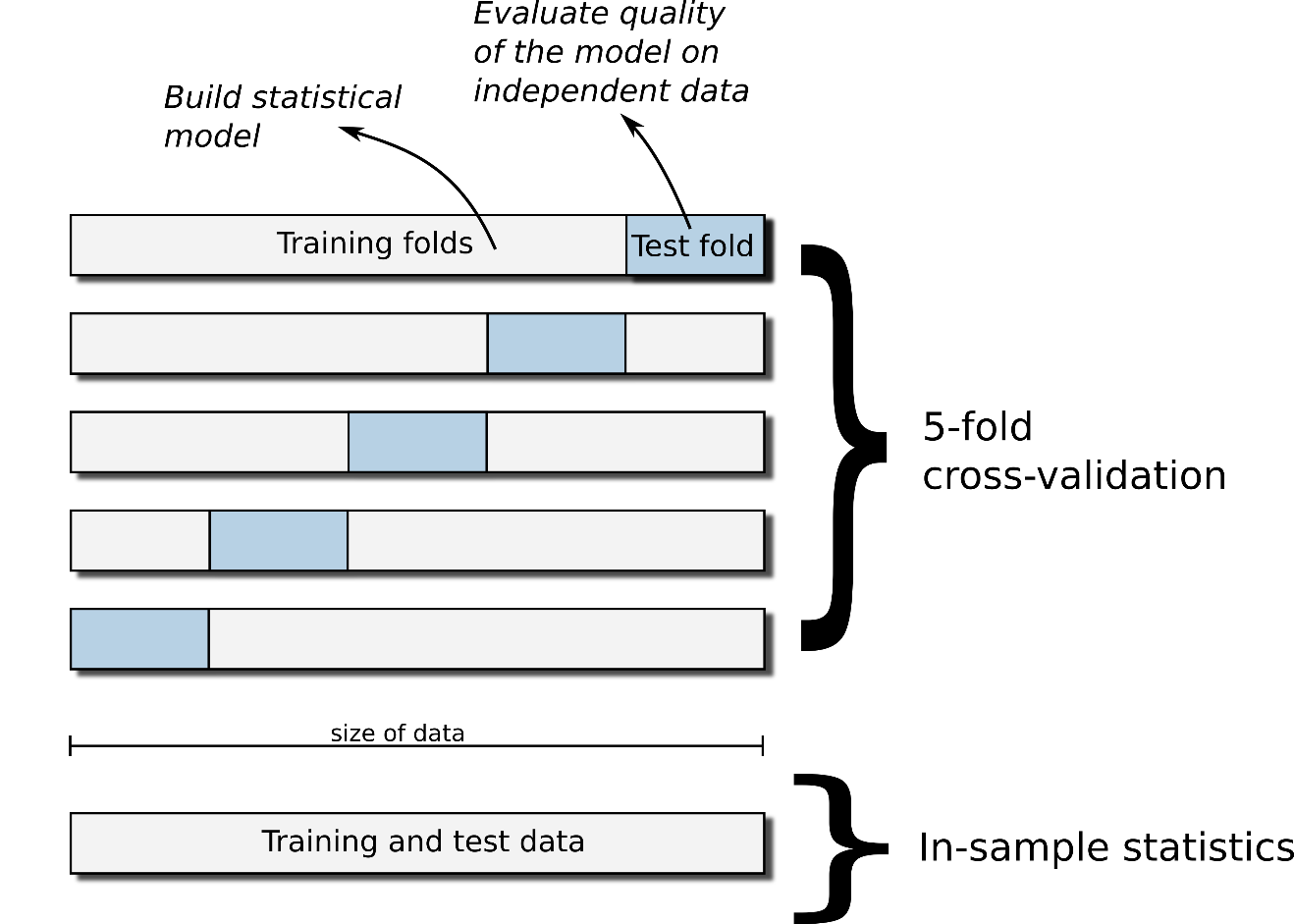


Machine learning techniques have found applications in regression and classification problems (Cristianini and Shawe-Taylor 2000) in psychiatric neuroimaging. In a recent review of machine learning in neuroimaging it was noted that, even though the so far used standard univariate analyses of neuroimaging data had revealed a significant amount of differences between healthy controls and patients suffering a wide range of psychiatric disorders, including Alzheimer's disease, schizophrenia, major depression and bipolar disorder, those analyses produced significant findings only at group level and had limited clinical translation. Therefore, alternative forms of analysis including support vector machines (SVMs), allowed developing classification algorithms on training data sets that enabled the categorization of an individual's previously unseen data into a predefined group (Orrù et al. 2012).

Examples provided by Iwabuchi et al. who tried to explore whether SVMs could be used as classifiers that could distinguish patients suffering from schizophrenia from healthy controls based on structural magnetic resonance data of brain grey and white matter morphometry (Iwabuchi, Liddle, and Palaniyappan 2013). Although this study had some limitations, like a relatively small study sample (n=39), their findings implied that machine learning could find applications in clinical settings and help clinical decision making if they could be widely applied. Other studies attempted to solve problems in psychiatry like identifying brain morphometry markers for the prognosis of depression (Nouretdinov et al. 2011), or depression classification and treatment response prediction(Patel et al. 2015), or improving the current screening methods for the diagnosis of autism (this paper used decision trees and a combinatory model of 16 different algorithms to produce an almost perfect sensitivity, specificity and accuracy, but with limitations) (Wall et al. 2012).

An interesting example was the attempt of predicting treatment response of clozapine therapy in patients with treatment resistant schizophrenia, using pre-treatment electroencephalography (EEG) readings. In a pilot study that used machine learning methodology quite intriguing results indicating the capacity to make predictions about the clinical response were found (Khodayari-Rostamabad et al. 2010), but a larger study that used traditional statistics did not find any significant results. The authors of the latter note that the results were disappointing, and expressed that a better methodology and study design might bear different results (Shrivastava et al. 2014). On the other hand, we have examples of successful replication of results of studies that used common statistical methods in re-analyses that used machine learning techniques. For example, event related potential (ERP) data, known to produce statistically significant differences between groups of infants that were categorized as low or high risk of developing autism later in life (Elsabbagh et al. 2009), were re-analysed and both the discriminant algorithmic approaches used were able to get above chance results between groups (Stahl et al. 2012).

While support vector machines (SMVs) are one of the most popular approaches, there are many different machine learning methods available. Random forests (Breiman 2001b) (RF) are known to handle noise well and they do not overfit. They have been proven to perform excellently in terms of accuracy with large numbers of predictor variables despite the presence of complex interactions (Robnik-Sikonja 2004). Those type of high-dimensional problems are common in psychiatry where large amounts of data are generated for each subject (Strobl, Malley, and Tutz 2009). Another reason why random forests might be useful in large data sets is their ability to make good use of compound variables. Compound variables derive from combining values, or bins of values to make a new variable. These compound variables are readily available from well-established factor analyses in the common questionnaires like the Barratt Impulsivity Scale (BIS) and the Padua Inventory (PI).

A comprehensive comparison between different machine learning algorithms is beyond the purpose of this report. A combinatory model of multiple algorithms where classification decisions would be made based on majority vote between models could have improved performance (Rose 2013), but this was not tested and considered beyond this proof-of-concept report.

# eMethods 2 – Data analysis details

Data variables and scores were loaded into the R-Studio workspace from the original database. All continuous variables including ASRS, BIS, PI questionnaires and Age were pre-processed (standardized) by subtracting the mean and dividing by the standard deviation. Binary and multi-level factor variables were used as originally coded: for Gender (1 = Male, 2 = Female), Race (1 = Caucasian, 2 = non-Caucasian, self-reported and then further categorized as a binary variable by the researchers), Education (1=<High School, 2=High School Graduates/General Education 3=Some College, 4=College Graduates, 5=Beyond College), Diagnostic variables for ADHD, OCD, GAD and SAD were used as factor variables (1 = diagnosis is present, 0 = diagnosis is absent). Diagnosis for ADHD was assessed with the ASRSv1.1 and the diagnoses for OCD, GAD and SAD were assessed with the MINI instrument.

A model prediction at the default ROC cut-off was used to calculate Accuracy, Sensitivity, Specificity, PPV, NPV, Kappa and F-measures. Those results were averaged over all replications and mean, standard deviation and standard error of the mean were calculated. Variable Importance matrices were averaged from all replications and re-arranged in order of descending importance. Another model prediction for classification probabilities was used to calculate ROC-AUC scores and ROC and PRC data points. ROC-AUC and PR-AUC scores were averaged over all replications and mean, standard deviation and standard error of the mean were calculated. ROC and PRC data points were respectively pooled to allow exploratory plotting of those results. ROC and PRC were plotted and a General Additive Model (GAM) line was fit with confidence intervals. A GAM line was chosen as there was no assumption of the shape (e.g. linear, quadratic or else) of the curves. Due to the way the plots were created those have only representational value. The mean ROC-AUC and PR-AUC scores are reported for the plots calculated as above. Variable importance (VI) was calculated for all included variables using the mean decrease of Gini, which is a parameter that essentially indicates how much a variable is used by an algorithm to help make classification decisions. VI results were averaged over the 50 replications and reported in descending order.

All five different variable sets that were tested used the same process as described above. These are the specific variables included in each set: (a) baseline variables included age, sex, race, education, social anxiety diagnosis and generalized anxiety disorder diagnosis, a total of 6 variables (b) impulsivity variables included Attention Impulsivity factor (BIS), Motor Impulsivity factor (BIS), Non-planning Impulsivity factor (BIS), ASRS, ADHD Diagnosis, a total of 5 variables (c) compulsivity variables included Padua Inventory Checking Compulsion, Padua Inventory Contamination Obsessions and Washing Compulsions, Padua Inventory Dressing and Grooming Compulsions, Padua Inventory Impulses to Harm Self or Others, Padua Inventory Thoughts of Harm to Self or Others and OCD Diagnosis, a total of six variables.

Finally, a different set was created by randomization of all variable scores in the full data set and creating a ‘random’ set. The random set included random variable scores of mean zero and standard deviation one for the continuous variables, random integers of zero and one for the binary variables and random integers equal to the number of factor levels for multi-level factor variables. Those sets were used for a ‘chance level’ baseline of predictions.

# eMethods 3 – Why report extra metrics?

Further metrics are reported in the supplement for completeness of presentation, however the authors do not rely on these metrics to draw conclusions for this report. Accuracy, most commonly reported in classification problems, is a skew sensitive metric that ignores the distribution of correct predictions over the classes; therefore it is problematic when used in unbalanced groups. Similarly, sensitivity, specificity, positive predictive value, negative predictive values and kappa scores assess specific aspects of the model’s goodness but have their own specific well known limitations.

One of the output measures of LR and RFs is the variable importance, which in simple terms describes how important is each variable for the algorithm to make decisions. Algorithms like random forests use decision trees with multiple branches. At each branch a variable score is used to make a classification prediction. Eventually many branches make a decision tree and many trees are averaged to create a random forest prediction. RFs can produce Variable Importance Matrices that are relatively intuitive to read and this can improve interpretation of results. As a simple example, if we had a classification problem trying to differentiate between apples and oranges, the variable ‘colour’ would have a high VI, the variable ‘shape’ would have a low VI and the variable ‘type of food’ i.e. ‘fruit’ on both cases, would have zero VI. That shows that some predictor variables (features) help the model making predictions more than others, and therefore are more important. LR variable importance matrices are less likely to improve interpretation of results as they become more unstable in the presence of co-linearity of predictor variables.

#

# eTable 1 – Demographic and clinical characteristics of the Stellenbosch sample

| **Variable** | **IAT score <50^a^** | **IAT score ≥ 50^b^** | **p-value** | **Corrected p-value (*217)^c^** | **Effect Size^d^** |
| --- | --- | --- | --- | --- | --- |
| **IAT scores** | 29.1 (7.3) | 60.9 (10.6) | <0.0001 v | <0.0001 v | 0.55 |
| **Age, years** | 26.4 (11.3) | 32.1 (15.1) | <0.0001 v | 0.0008 v | 0.02 |
| **Gender, male, n (%)** | 752 (61.8) | 59 (59.0) | 0.6492 | >0.99 |  |
| **Race, Caucasian, n (%)** | 875 (71.9) | 51 (51.0) | <0.0001 | 0.0037 | 0.12 |
| **Education, n (%)**  **Below high school** | 1 (0.0) | 0 (0) |  |  |  |
| **High school graduate** | 147 (12.0) | 16 (16.0) |  |  |  |
| **Some college** | 248 (20.3) | 34 (34.0) | 0.0079 | >0.99 |  |
| **College graduate** | 518 (42.6) | 34 (34.0) |  |  |  |
| **Beyond College** | 302 (24.8) | 16 (16.0) |  |  |  |
| **GAD, n (%)** | 203 (16.7) | 33 (33.0) | <0.0001 | 0.0169 | 0.11 |
| **Social Anxiety Disorder, n (%)** | 144 (11.8) | 26 (26.0) | <0.0001 | 0.0207 | 0.11 |
| **ADHD, n (%)** | 538 (44.2) | 65 (65.0) | <0.0001 | 0.0208 | 0.11 |
| **OCD, n (%)** | 122 (10.0) | 25 (25.0) | <0.0001 | 0.0023 | 0.13 |

**^a^** IAT score <50 (Controls Stellenbosch, South Africa n = 1216);

**^b^** IAT score ≥ 50 (problematic internet use Stellenbosch, South Africa n = 100);

All scores are mean (SD) unless otherwise noted. Statistic: chi-square except where indicated with ‘v’ ANOVAs for. Numbers in parentheses are percentages of each element in the respective groups. GAD: Generalized Anxiety Disorder; ADHD: Attention-Deficit Hyperactivity Disorder; OCD: Obsessive-Compulsive Disorder

**^c^** Bonferroni correction applied *217

**^d^** Effect sizes are eta squared for ANOVA and phi for chi square tests

# eTable 2 – Demographic and clinical characteristics of the Chicago sample

| **Variable** | **IAT score <50^a^** | **IAT score ≥ 50^b^** | **p-value** | **Corrected p-value (*217)^c^** | **Effect Size^d^** |
| --- | --- | --- | --- | --- | --- |
| **IAT scores** | 32.9 (6.8) | 58.62 (8.5) | <0.0001 v | <0.0001 v | 0.57 |
| **Age, years** | 36.5 (14.5) | 34.7 (13.2) | 0.303 v | >0.99 v |  |
| **Gender, male, n (%)** | 447 (73.3) | 58 (71.6) | >0.99 | >0.99 |  |
| **Race, Caucasian, n (%)** | 470 (77.2) | 51 (62.9) | 0.0662 | >0.99 |  |
| **Education, n (%)**  **Below high school** | 11 (1.8) | 1 (1.1) |  |  |  |
| **High school graduate** | 51 (8.3) | 10 (12.3) |  |  |  |
| **Some college** | 196 (32.1) | 34 (41.9) | 0.0568 | >0.99 |  |
| **College graduate** | 222 (36.4) | 29 (35.8) |  |  |  |
| **Beyond College** | 129 (21.1) | 7 (8.6) |  |  |  |
| **GAD, n (%)** | 119 (19.5) | 45 (55.5) | <0.0001 | <0.0001 | 0.26 |
| **Social Anxiety Disorder, n (%)** | 65 (10.6) | 32 (39.5) | <0.0001 | <0.0001 | 0.26 |
| **ADHD, n (%)** | 215 (35.3) | 66 (81.4) | <0.0001 | <0.0001 | 0.29 |
| **OCD, n (%)** | 37 (6.1) | 25 (30.8) | <0.0001 | <0.0001 | 0.26 |

**^a^** IAT score <50 (Controls Chicago n = 609)

**^b^** IAT score ≥ 50 (problematic internet use Chicago n = 81);

All scores are mean (SD) unless otherwise noted. Statistic: chi-square except where indicated with ‘v’ ANOVAs for. Numbers in parentheses are percentages of each element in the respective groups. GAD: Generalized Anxiety Disorder; ADHD: Attention-Deficit Hyperactivity Disorder; OCD: Obsessive-Compulsive Disorder

**^c^** Bonferroni correction applied *217

**^d^** Effect sizes are eta squared for ANOVA and phi for chi square tests

# eTable 3 - Summary of comparisons between models that include impulsivity or compulsivity measures against models with baseline variables only

|  |  | **Logistic regression** | | **Random Forest** | | **Naïve Bayes** | |
| --- | --- | --- | --- | --- | --- | --- | --- |
| **Validation Set-up** | **Variables included in the model** | **Comparison vs ROC-AUC mean^a^** | **Comparison vs PR-AUC mean^a^** | **Comparison vs ROC-AUC mean^a^** | **Comparison vs PR-AUC mean^a^** | **Comparison vs ROC-AUC mean^a^** | **Comparison vs PR-AUC mean^a^** |
| **Full data set-up (A)** | **Impulsivity only** | <0.001 | <0.001 | <0.001 | <0.001 | <0.001 | <0.001 |
|  | **Compulsivity only** | <0.001 | <0.001 | <0.001 | 0.0002 | <0.001 | <0.001 |
| **Stellenbosch set-up (B)** | **Impulsivity only** | >0.99 | >0.99 | <0.001 | 0.11 | <0.001 | <0.001 |
|  | **Compulsivity only** | <0.001 | <0.001 | <0.001 | <0.001 | <0.001 | <0.001 |
| **Chicago set-up (C)** | **Impulsivity only** | <0.001 | <0.001 | <0.001 | <0.001 | <0.001 | <0.001 |
|  | **Compulsivity only** | <0.001 | <0.001 | <0.001 | <0.001 | <0.001 | <0.001 |
| **Validation Set up (D)^b^** | **Impulsivity only** | <0.001 | <0.001 | <0.001 | >0.99 | <0.001 | <0.001 |
|  | **Compulsivity only** | <0.001 | <0.001 | <0.001 | <0.001 | <0.001 | <0.001 |
| **Validation set-up (E) ^c^** | **Impulsivity only** | <0.001 | <0.001 | <0.001 | <0.001 | <0.001 | <0.001 |
|  | **Compulsivity only** | <0.001 | <0.001 | <0.001 | <0.001 | <0.001 | <0.001 |

All p values are Wilcoxon signed rank test with continuity correction. All significant values support the alternative hypothesis that true difference in means is not equal to zero. In all comparisons the baseline ROC-AUC and PR-AUC means were lower.

ROC-AUC: Receiver-operating characteristic Curve – Area Under the curve, PR-AUC: Precision-Recall curve – Area under the curve, SD: standard deviation

**^a^** Baseline model with only demographics and anxiety disorder diagnoses.

^b^ Training the model in the Stellenbosch set and testing on the Chicago set

^c^ Training the model in the Chicago set and testing on the Stellenbosch set

All p values are Bonferroni corrected for multiple comparisons *217

# eTable 4 - Summary of comparisons between models that include both impulsivity and compulsivity measures against models with impulsivity or compulsivity only

|  |  | **Logistic regression** | | **Random Forest** | | **Naïve Bayes** | |
| --- | --- | --- | --- | --- | --- | --- | --- |
| **Validation Set-up** | **Variables included in the model** | **Comparison vs ROC-AUC mean^a^** | **Comparison vs PR-AUC mean^a^** | **Comparison vs ROC-AUC mean^a^** | **Comparison vs PR-AUC mean^a^** | **Comparison vs ROC-AUC mean^a^** | **Comparison vs PR-AUC mean^a^** |
| **Full data set-up (A)** | **Impulsivity only** | <0.001 | <0.001 | <0.001 | <0.001 | <0.001 | <0.001 |
|  | **Compulsivity only** | <0.001 | <0.001 | <0.001 | 0.18 | <0.001 | <0.001 |
| **Stellenbosch set-up (B)** | **Impulsivity only** | <0.001 | <0.001 | <0.001 | 0.036 | <0.001 | <0.001 |
|  | **Compulsivity only** | >0.99 | >0.99 | <0.001 | >0.99 | <0.001 | >0.99 |
| **Chicago set-up (C)** | **Impulsivity only** | >0.99 | <0.001 | 0.0018 | <0.001 | >0.99 | <0.001 |
|  | **Compulsivity only** | <0.001 | <0.001 | <0.001 | 0.44 | <0.001 | <0.001 |
| **Validation Set up (D)^b^** | **Impulsivity only** | <0.001 | <0.001 | <0.001 | <0.001 | <0.001 | <0.001 |
|  | **Compulsivity only** | <0.001 | <0.001 | <0.001 | >0.99 | <0.001 | <0.001 |
| **Validation set-up (E) ^c^** | **Impulsivity only** | <0.001 | <0.001 | <0.001 | <0.001 | <0.001 | <0.001 |
|  | **Compulsivity only** | <0.001 | <0.001 | <0.001 | >0.99 | <0.001 | <0.001 |

All p values are Wilcoxon signed rank test with continuity correction. All significant values support the alternative hypothesis that true location shift is not equal to zero and therefore models that included both impulsivity and compulsivity were superior to models with impulsivity or compulsivity only. ROC-AUC: Receiver-operating characteristic Curve – Area Under the curve, PR-AUC: Precision-Recall curve – Area under the curve, SD: standard deviation

**^a^** Model with baseline and both impulsivity and compulsivity variables

^b^ Training the model in the Stellenbosch set and testing on the Chicago set

^c^ Training the model in the Chicago set and testing on the Stellenbosch set

All p values are Bonferroni corrected for multiple comparisons *217

# eTable 5 – Head-to-head comparisons between Logistic Regression and Random Forests that include both impulsivity and compulsivity measures

| **Validation Set-up** | **ROC-AUC** | **PR-AUC** |
| --- | --- | --- |
| **Full data set-up (A)** | >0.99 | <0.0001  (LR superiority) |
| **Stellenbosch set-up (B)** | >0.99 | <0.0001  (LR superiority) |
| **Chicago set-up (C)** | 0.0006  (RF superiority) | <0.0001  (LR superiority) |
| **Validation Set up (D)^b^** | >0.99 | <0.0001  (LR superiority) |
| **Validation set-up (E) ^c^** | <0.001  (LR superiority) | <0.0001  (LR superiority) |

All p values are Wilcoxon signed rank test with continuity correction. All significant values support the alternative hypothesis that true location shift is not equal to zero. Superiority of models in each comparison is indicated in parentheses. ROC-AUC: Receiver-operating characteristic Curve – Area Under the curve; PR-AUC - Precision-Recall curve – Area under the curve, SD - standard deviation; LR – Logistic Regression; RF – Random Forests; NB – Naïve Bayes

**^a^** Model with baseline and both impulsivity and compulsivity variables

^b^ Training the model in the Stellenbosch set and testing on the Chicago set

^c^ Training the model in the Chicago set and testing on the Stellenbosch set

All p values are Bonferroni corrected for multiple comparisons *217

# eTable 6 – Head-to-head comparisons between Logistic Regression and Naïve Bayes models that include both impulsivity and compulsivity measures

| **Validation Set-up** | **ROC-AUC** | **PR-AUC** |
| --- | --- | --- |
| **Full data set-up (A)** | >0.99 | >0.99 |
| **Stellenbosch set-up (B)** | >0.99 | <0.0001  (LR superiority) |
| **Chicago set-up (C)** | 0.0104  (NB superiority) | <0.0001  (NB superiority) |
| **Validation Set up (D)^b^** | <0.0001  (NB superiority) | <0.0001  (LR superiority) |
| **Validation set-up (E) ^c^** | >0.99 | <0.0001  (NB superiority) |

All p values are Wilcoxon signed rank test with continuity correction. All significant values support the alternative hypothesis that true location shift is not equal to zero. Superiority of models in each comparison is indicated in parentheses. ROC-AUC: Receiver-operating characteristic Curve – Area Under the curve; PR-AUC: Precision-Recall curve – Area under the curve; SD – standard deviation; LR – Logistic Regression; RF – Random Forests; NB – Naïve Bayes

**^a^** Model with baseline and both impulsivity and compulsivity variables

^b^ Training the model in the Stellenbosch set and testing on the Chicago set

^c^ Training the model in the Chicago set and testing on the Stellenbosch set

All p values are Bonferroni corrected for multiple comparisons *217

# eTable 7 – Head-to-head comparisons between Random Forests and Naïve Bayes models that include both impulsivity and compulsivity measures

| **Validation Set-up** | **ROC-AUC** | **PR-AUC** |
| --- | --- | --- |
| **Full data set-up (A)** | >0.99 | <0.0001  (NB superiority) |
| **Stellenbosch set-up (B)** | >0.99 | 0.0013  (RF superiority) |
| **Chicago set-up (C)** | >0.99 | <0.0001  (NB superiority) |
| **Validation Set up (D)^b^** | <0.001  (NB superiority) | <0.0001  (RF superiority) |
| **Validation set-up (E) ^c^** | <0.001  (NB superiority) | <0.0001  (NB superiority) |

All p values are Wilcoxon signed rank test with continuity correction. All significant values support the alternative hypothesis that true location shift is not equal to zero. Superiority of models in each comparison is indicated in parentheses. ROC-AUC: Receiver-operating characteristic Curve – Area Under the curve; PR-AUC - Precision-Recall curve – Area under the curve, SD - standard deviation; LR – Logistic Regression; RF – Random Forests; NB – Naïve Bayes

**^a^** Model with baseline and both impulsivity and compulsivity variables

^b^ Training the model in the Stellenbosch set and testing on the Chicago set

^c^ Training the model in the Chicago set and testing on the Stellenbosch set

All p values are Bonferroni corrected for multiple comparisons *217

# eTable 8: Validation set-up (A) Full data basic metrics for all models

| **Model** | **Metrics** | **IMP-COMP set mean ± SD** | **IMP only set mean ± SD** | **COMP only set mean ± SD** | **Baseline set mean ± SD** | **‘Chance level’ set** |
| --- | --- | --- | --- | --- | --- | --- |
| **Logistic Regression** | **ROC-AUC** | 0.83 ± 0.031 | 0.79 ± 0.033 | 0.80 ± 0.033 | 0.73 ± 0.034 | 0.50 ± 0.034 |
|  | **PR-AUC** | 0.26 ± 0.036 | 0.17 ± 0.021 | 0.23 ± 0.041 | 0.10 ± 0.016 | 0.02 ± 0.002 |
|  | **Accuracy** | 0.92 ± 0.006 | 0.91 ± 0.005 | 0.91 ± 0.007 | 0.91 ± 0.003 | 0.91 ± 0.003 |
|  | **Sensitivity** | 0.64 ± 0.123 | 0.56 ± 0.183 | 0.61 ± 0.169 | 0.60 ± 0.336 | 0.00 ± 0.000 |
|  | **Specificity** | 0.93 ± 0.005 | 0.92 ± 0.003 | 0.92 ± 0.005 | 0.91 ± 0.001 | 0.91 ± 0.000 |
|  | **PPV** | 0.21 ± 0.055 | 0.10 ± 0.034 | 0.16 ± 0.056 | 0.03 ± 0.020 | 0.00 ± 0.000 |
|  | **NPV** | 0.99 ± 0.006 | 0.99 ± 0.005 | 0.99 ± 0.006 | 0.99 ± 0.002 | 1.00 ± 0.000 |
|  | **Kappa** | 0.92 ± 0.006 | 0.91 ± 0.005 | 0.91 ± 0.007 | 0.91 ± 0.003 | 0.91 ± 0.000 |
|  | **F Measure** | 0.34 ± 0.074 | 0.18 ± 0.056 | 0.27 ± 0.082 | 0.05 ± 0.037 | 0.00 ± 0.000 |
| **Random Forests** | **ROC-AUC** | 0.84 ± 0.026 | 0.80 ± 0.026 | 0.79 ± 0.034 | 0.69 ± 0.035 | 0.51 ± 0.038 |
|  | **PR-AUC** | 0.20 ± 0.026 | 0.12 ± 0.023 | 0.18 ± 0.030 | 0.10 ± 0.052 | 0.04 ± 0.012 |
|  | **Accuracy** | 0.92 ± 0.005 | 0.91 ± 0.002 | 0.91 ± 0.004 | 0.91 ± 0.009 | 0.91 ± 0.002 |
|  | **Sensitivity** | 0.63 ± 0.149 | 0.52 ± 0.397 | 0.72 ± 0.213 | 0.38 ± 0.175 | 0.00 ± 0.000 |
|  | **Specificity** | 0.92 ± 0.005 | 0.91 ± 0.002 | 0.92 ± 0.004 | 0.91 ± 0.005 | 0.91 ± 0.000 |
|  | **PPV** | 0.14 ± 0.056 | 0.02 ± 0.028 | 0.10 ± 0.053 | 0.06 ± 0.070 | 0.00 ± 0.008 |
|  | **NPV** | 0.99 ± 0.005 | 0.99 ± 0.002 | 0.99 ± 0.005 | 0.99 ± 0.014 | 0.99 ± 0.002 |
|  | **Kappa** | 0.91 ± 0.005 | 0.91 ± 0.002 | 0.91 ± 0.004 | 0.91 ± 0.009 | 0.91 ± 0.002 |
|  | **F Measure** | 0.24 ± 0.084 | 0.03 ± 0.050 | 0.18 ± 0.084 | 0.10 ± 0.120 | 0.01 ± 0.043 |
| **Naïve Bayes** | **ROC-AUC** | 0.83 ± 0.030 | 0.80 ± 0.032 | 0.79 ± 0.033 | 0.74 ± 0.035 | 0.50 ± 0.038 |
|  | **PR-AUC** | 0.25 ± 0.051 | 0.04 ± 0.020 | 0.17 ± 0.046 | 0.00 ± 0.001 | 0.02 ± 0.001 |
|  | **Accuracy** | 0.92 ± 0.005 | 0.91 ± 0.001 | 0.91 ± 0.005 | 0.91 ± 0.000 | 0.91 ± 0.001 |
|  | **Sensitivity** | 0.65 ± 0.138 | 0.78 ± 0.334 | 0.66 ± 0.196 | 0.00 ± 0.000 | 0.00 ± 0.000 |
|  | **Specificity** | 0.92 ± 0.004 | 0.91 ± 0.001 | 0.92 ± 0.003 | 0.91 ± 0.000 | 0.91 ± 0.001 |
|  | **PPV** | 0.15 ± 0.053 | 0.01 ± 0.001 | 0.09 ± 0.034 | 0.00 ± 0.000 | 0.00 ± 0.000 |
|  | **NPV** | 0.99 ± 0.004 | 1.00 ± 0.011 | 0.99 ± 0.004 | 1.00 ± 0.000 | 1.00 ± 0.001 |
|  | **Kappa** | 0.92 ± 0.005 | 0.91 ± 0.001 | 0.91 ± 0.005 | 0.91 ± 0.000 | 0.91 ± 0.001 |
|  | **F Measure** | 0.25 ± 0.078 | 0.01 ± 0.001 | 0.17 ± 0.056 | 0.00 ± 0.000 | 0.00 ± 0.000 |

**AUC** – Area under the curve; **COMP only** – Prediction model using baseline plus compulsivity variables; **F Measure** – balanced F-score (*F_1_*); **IMP only** – Prediction model using baseline plus impulsivity variables; **IMP-COMP** – Prediction model using baseline plus impulsivity-compulsivity variables; **Kappa** – Cohen's kappa coefficient; **NPV** – Negative predictive value; **PPV** – Positive predictive value; **PR** – Precision-Recall (PRC – Precision-Recall Curve); **ROC** – Receiver Operating Characteristic curve; **SD** – Standard deviation

# eTable 9: Validation set-up (A) Full data Variable Importance matrices from prediction using baseline plus impulsivity-compulsivity variables of Logistic Regression and Random Forests

|  | **Logistic Regression** | | **Random Forest** | |
| --- | --- | --- | --- | --- |
| **Rank** | **Variable** | **VI %** | **Variable** | **VI %** |
| 1 | Age (older) | 99.0 | ASRS | 95.0 |
| 2 | Race (non-Caucasian) | 78.0 | Age (older) | 92.0 |
| 3 | PI-IHSO | 74.0 | PI-CC | 91.0 |
| 4 | PI-CC | 62.0 | PI-ISHO | 90.0 |
| 5 | BISMI | 61.0 | BISMI | 79.0 |
| 6 | ASRS | 41.0 | PI-THSO | 75.0 |
| 7 | GAD DIAGNOSIS | 28.0 | BISNI | 75.0 |
| 8 | ADHD DIAGNOSIS | 28.0 | BISAI | 68.0 |
| 9 | PI-DGC | 25.0 | PI-COWC | 67.0 |
| 10 | BISAI | 21.0 | PI-DGC | 60.0 |
| 11 | Social Anxiety Diagnosis | 15.0 | Race | 20.0 |
| 12 | OCD DIAGNOSIS | 14.0 | GAD DIAGNOSIS | 15.0 |
| 13 | Gender (Male) | 12.0 | OCD DIAGNOSIS | 15.0 |
| 14 | BISNI | 11.0 | Social Anxiety Diagnosis | 14.0 |
| 15 | PI-THSO | 8.5 | Education (Some College) | 14.0 |
| 16 | PI-COWC | 7.5 | ADHD DIAGNOSIS | 13.0 |
| 17 | Education (Some College) | 7.3 | Gender | 12.0 |
| 18 | Education (High School) | 7.1 | Education (College) | 11.0 |
| 19 | Education (College) | 5.1 | Education (High School) | 9.6 |
| 20 | Education (Beyond College) | 4.3 | Education (Beyond College) | 7.4 |

**ADHD –** Attention Deficit Hyperactivity Disorder**, ASRS** - Adult ADHD Self-Report Scale (ASRS-v1.1), **BISAI** - Barratt Impulsiveness Scale 11 Attention Impulsivity factor (BIS), **BISMI** - Barratt Impulsiveness Scale 11 Motor Impulsivity factor (BIS), **BISNI** - Barratt Impulsiveness Scale 11 Non-planning Impulsivity factor (BIS), **GAD** – Generalized Anxiety disorder, **OCD** – Obsessive-Compulsive disorder, **PI-CC** – Padua Inventory-Revised Checking Compulsion, **PI-COWC** – Padua Inventory-Revised Contamination Obsessions and Washing Compulsions, **PI-DGC** – Padua Inventory-Revised Dressing and Grooming Compulsions, **PI-IHSO** – Padua Inventory-Revised Impulses to Harm Self or Others, **PI-THSO** – Padua Inventory-Revised Thoughts of Harm to Self or Others

# eFigure 1: Validation set-up (A) Full data Receiver Operating Characteristic and Precision-Recall Curves for all models


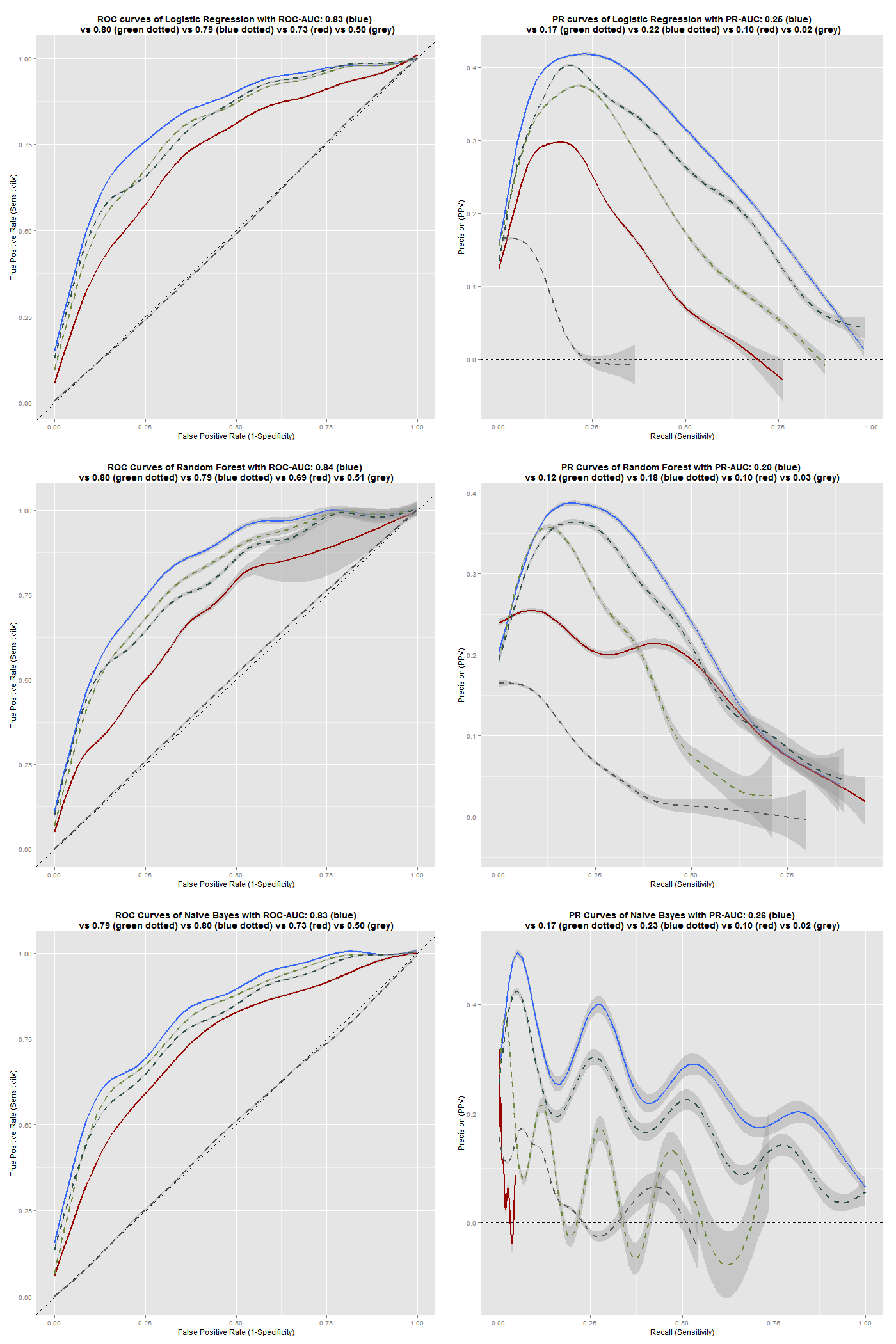


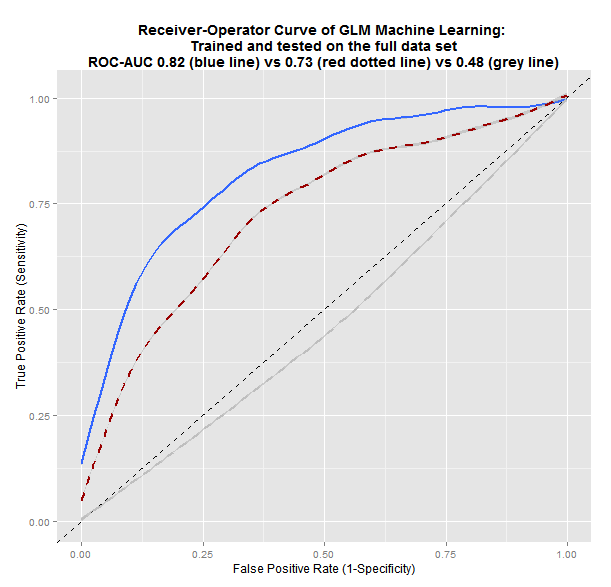
 Prediction model curve using baseline plus impulsivity and compulsivity variables


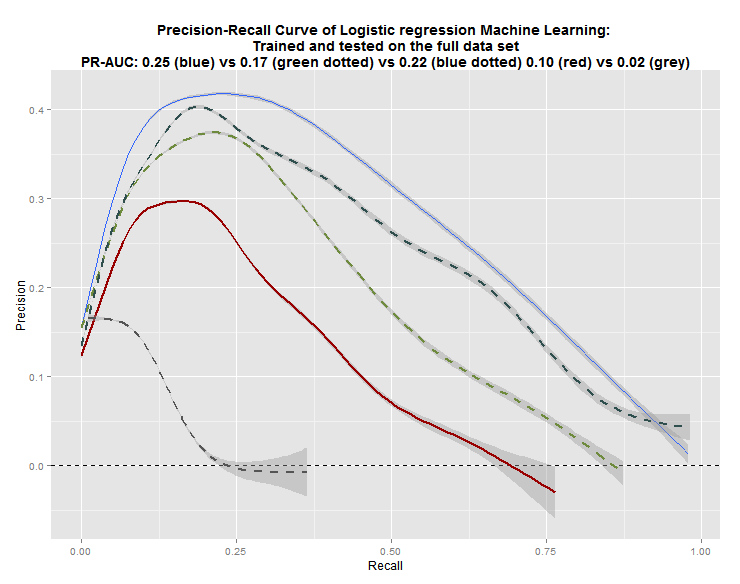
 Prediction model curve using baseline plus impulsivity variables


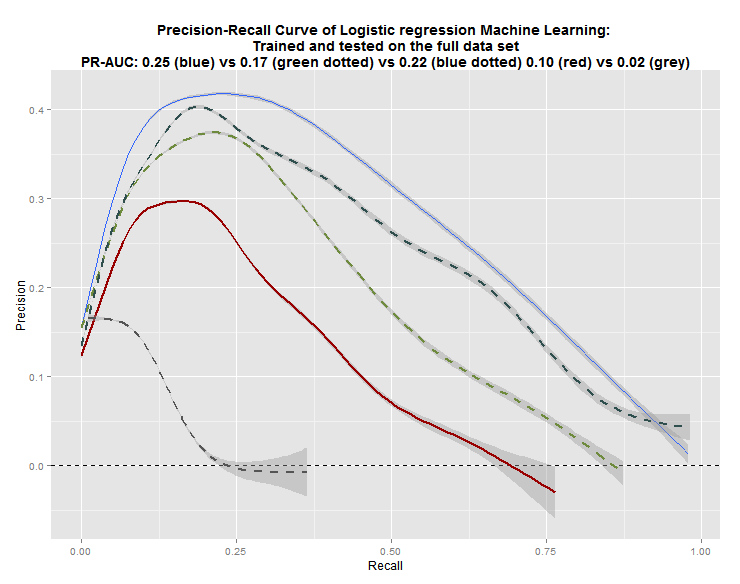
 Prediction model curve using baseline plus compulsivity variables


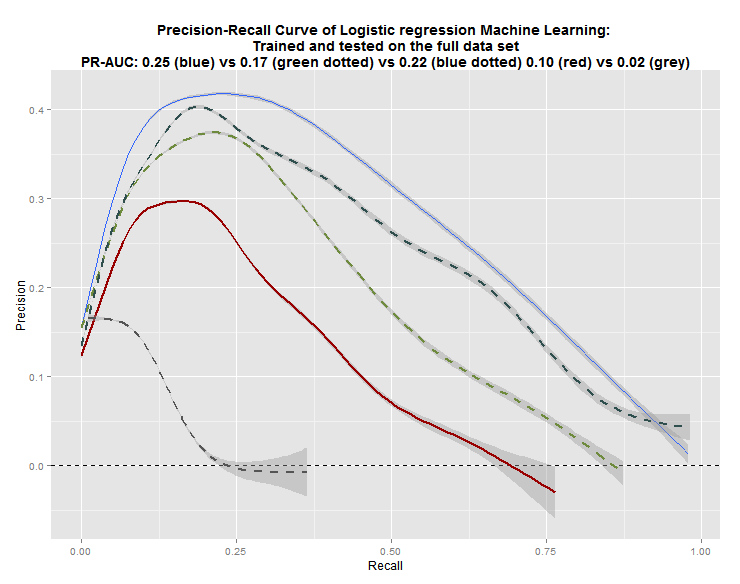
 Prediction model curve using baseline variables only


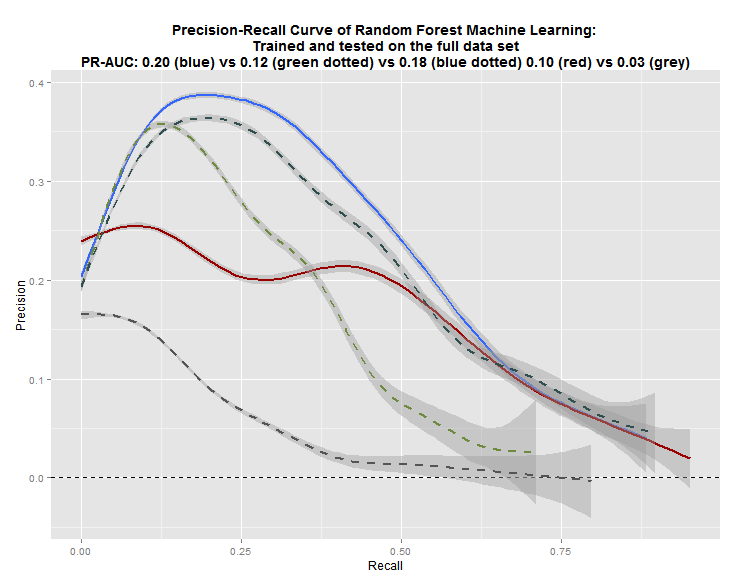
 Prediction model curve ‘at chance’ level with randomized variable scores

# eTable 10: Validation set-up (B) Stellenbosch set basic metrics for all models

| **Model** | **Metrics** | **IMP-COMP set mean ± SD** | **IMP only set mean ± SD** | **COMP only set mean ± SD** | **Baseline set mean ± SD** | **‘Chance level’ set** |
| --- | --- | --- | --- | --- | --- | --- |
| **Logistic Regression** | **ROC-AUC** | 0.80 ± 0.034 | 0.73 ± 0.041 | 0.80 ± 0.033 | 0.79 ± 0.036 | 0.52 ± 0.044 |
|  | **PR-AUC** | 0.21 ± 0.043 | 0.10 ± 0.022 | 0.23 ± 0.041 | 0.21 ± 0.043 | 0.02 ± 0.003 |
|  | **Accuracy** | 0.93 ± 0.006 | 0.92 ± 0.004 | 0.91 ± 0.007 | 0.93 ± 0.007 | 0.92 ± 0.000 |
|  | **Sensitivity** | 0.64 ± 0.182 | 0.36 ± 0.340 | 0.61 ± 0.169 | 0.65 ± 0.200 | 0.00 ± 0.000 |
|  | **Specificity** | 0.94 ± 0.004 | 0.93 ± 0.002 | 0.92 ± 0.005 | 0.93 ± 0.004 | 0.92 ± 0.000 |
|  | **PPV** | 0.16 ± 0.060 | 0.03 ± 0.035 | 0.16 ± 0.056 | 0.15 ± 0.057 | 0.00 ± 0.000 |
|  | **NPV** | 0.99 ± 0.006 | 0.99 ± 0.004 | 0.99 ± 0.006 | 0.99 ± 0.006 | 1.00 ± 0.000 |
|  | **Kappa** | 0.93 ± 0.006 | 0.92 ± 0.004 | 0.91 ± 0.007 | 0.93 ± 0.007 | 0.92 ± 0.000 |
|  | **F Measure** | 0.26 ± 0.087 | 0.06 ± 0.063 | 0.27 ± 0.082 | 0.26 ± 0.084 | 0.00 ± 0.000 |
| **Random Forests** | **ROC-AUC** | 0.81 ± 0.033 | 0.76 ± 0.042 | 0.75 ± 0.045 | 0.69 ± 0.042 | 0.53 ± 0.051 |
|  | **PR-AUC** | 0.14 ± 0.038 | 0.11 ± 0.034 | 0.15 ± 0.038 | 0.08 ± 0.041 | 0.03 ± 0.072 |
|  | **Accuracy** | 0.93 ± 0.005 | 0.92 ± 0.006 | 0.93 ± 0.006 | 0.91 ± 0.011 | 0.92 ± 0.003 |
|  | **Sensitivity** | 0.60 ± 0.258 | 0.33 ± 0.285 | 0.63 ± 0.291 | 0.23 ± 0.163 | 0.00 ± 0.000 |
|  | **Specificity** | 0.93 ± 0.005 | 0.93 ± 0.003 | 0.93 ± 0.005 | 0.92 ± 0.003 | 0.92 ± 0.001 |
|  | **PPV** | 0.08 ± 0.068 | 0.03 ± 0.046 | 0.10 ± 0.067 | 0.06 ± 0.054 | 0.00 ± 0.008 |
|  | **NPV** | 0.99 ± 0.005 | 0.99 ± 0.007 | 0.99 ± 0.006 | 0.98 ± 0.014 | 0.99 ± 0.003 |
|  | **Kappa** | 0.93 ± 0.005 | 0.92 ± 0.006 | 0.93 ± 0.006 | 0.91 ± 0.011 | 0.92 ± 0.003 |
|  | **F Measure** | 0.13 ± 0.120 | 0.06 ± 0.081 | 0.18 ± 0.011 | 0.10 ± 0.093 | 0.00 ± 0.015 |
| **Naïve Bayes** | **ROC-AUC** | 0.80 ± 0.034 | 0.75 ± 0.044 | 0.77 ± 0.037 | 0.71 ± 0.044 | 0.49 ± 0.042 |
|  | **PR-AUC** | 0.10 ± 0.054 | 0.06 ± 0.004 | 0.10 ± 0.054 | 0.00 ± 0.001 | 0.00 ± 0.001 |
|  | **Accuracy** | 0.93 ± 0.003 | 0.92 ± 0.000 | 0.92 ± 0.004 | 0.92 ± 0.000 | 0.92 ± 0.000 |
|  | **Sensitivity** | 0.58 ± 0.332 | 0.00 ± 0.000 | 0.55 ± 0.334 | 0.00 ± 0.000 | 0.00 ± 0.000 |
|  | **Specificity** | 0.93 ± 0.003 | 0.92 ± 0.000 | 0.93 ± 0.002 | 0.92 ± 0.000 | 0.92 ± 0.000 |
|  | **PPV** | 0.04 ± 0.038 | 0.00 ± 0.000 | 0.04 ± 0.032 | 0.00 ± 0.000 | 0.00 ± 0.000 |
|  | **NPV** | 0.99 ± 0.003 | 1.00 ± 0.000 | 0.99 ± 0.003 | 1.00 ± 0.000 | 1.00 ± 0.000 |
|  | **Kappa** | 0.93 ± 0.003 | 0.92 ± 0.000 | 0.92 ± 0.004 | 0.92 ± 0.000 | 0.92 ± 0.000 |
|  | **F Measure** | 0.08 ± 0.067 | 0.00 ± 0.000 | 0.07 ± 0.058 | 0.00 ± 0.000 | 0.00 ± 0.000 |

**AUC** – Area under the curve; **COMP only** – Prediction model using baseline plus compulsivity variables; **F Measure** – balanced F-score (*F_1_*); **IMP only** – Prediction model using baseline plus impulsivity variables; **IMP-COMP** – Prediction model using baseline plus impulsivity-compulsivity variables; **Kappa** – Cohen's kappa coefficient; **NPV** – Negative predictive value; **PPV** – Positive predictive value; **PR** – Precision-Recall (PRC – Precision-Recall Curve); **ROC** – Receiver Operating Characteristic curve; **SD** – Standard deviation

# eTable 11: Validation set-up (B) Stellenbosch set Variable Importance matrices from prediction using baseline plus impulsivity-compulsivity variables of Logistic Regression and Random Forests

|  | **Logistic Regression** | | **Random Forest** | |
| --- | --- | --- | --- | --- |
| **Rank** | **Variable** | **VI %** | **Variable** | **VI %** |
| 1 | Age (older) | 100.0 | PI-IHSO | 95.0 |
| 2 | PI-IHSO | 72.0 | Age (older) | 92.0 |
| 3 | Race (non-Caucasian) | 66.0 | ASRS | 89.0 |
| 4 | PI-CC | 48.0 | PI-CC | 82.0 |
| 5 | PI-DGC | 39.0 | PI-THSO | 68.0 |
| 6 | ADHD DIAGNOSIS | 29.0 | PI-COWC | 68.0 |
| 7 | BISMI | 26.0 | BISMI | 67.0 |
| 8 | PI-COWC | 23.0 | BISNI | 65.0 |
| 9 | PI-THSO | 22.0 | PI-DGC | 62.0 |
| 10 | BISNI | 18.0 | BISAI | 60.0 |
| 11 | GAD DIAGNOSIS | 14.0 | Race (non-Caucasian) | 21.0 |
| 12 | Education (Some College) | 11.0 | OCD DIAGNOSIS | 13.0 |
| 13 | ASRS | 11.0 | Gender (Male) | 12.0 |
| 14 | BISAI | 11.0 | Education (Some College) | 12.0 |
| 15 | Gender (Male) | 9.3 | ADHD DIAGNOSIS | 12.0 |
| 16 | Education (High School) | 8.9 | GAD DIAGNOSIS | 12.0 |
| 17 | OCD DIAGNOSIS | 8.8 | Social Anxiety Diagnosis | 11.0 |
| 18 | Social Anxiety Diagnosis | 8.6 | Education (College) | 10.0 |
| 19 | Education (College) | 4.0 | Education (High School) | 9.3 |
| 20 | Education (Beyond College) | 0.02 | Education (Beyond College) | 7.9 |

**ADHD –** Attention Deficit Hyperactivity Disorder**, ASRS** - Adult ADHD Self-Report Scale (ASRS-v1.1), **BISAI** - Barratt Impulsiveness Scale 11 Attention Impulsivity factor (BIS), **BISMI** - Barratt Impulsiveness Scale 11 Motor Impulsivity factor (BIS), **BISNI** - Barratt Impulsiveness Scale 11 Non-planning Impulsivity factor (BIS), **GAD** – Generalized Anxiety disorder, **OCD** – Obsessive-Compulsive disorder, **PI-CC** – Padua Inventory-Revised Checking Compulsion, **PI-COWC** – Padua Inventory-Revised Contamination Obsessions and Washing Compulsions, **PI-DGC** – Padua Inventory-Revised Dressing and Grooming Compulsions, **PI-IHSO** – Padua Inventory-Revised Impulses to Harm Self or Others, **PI-THSO** – Padua Inventory-Revised Thoughts of Harm to Self or Others

# eFigure 2: Validation set-up (B) Stellenbosch set Receiver Operating Characteristic and Precision-Recall Curves for all models


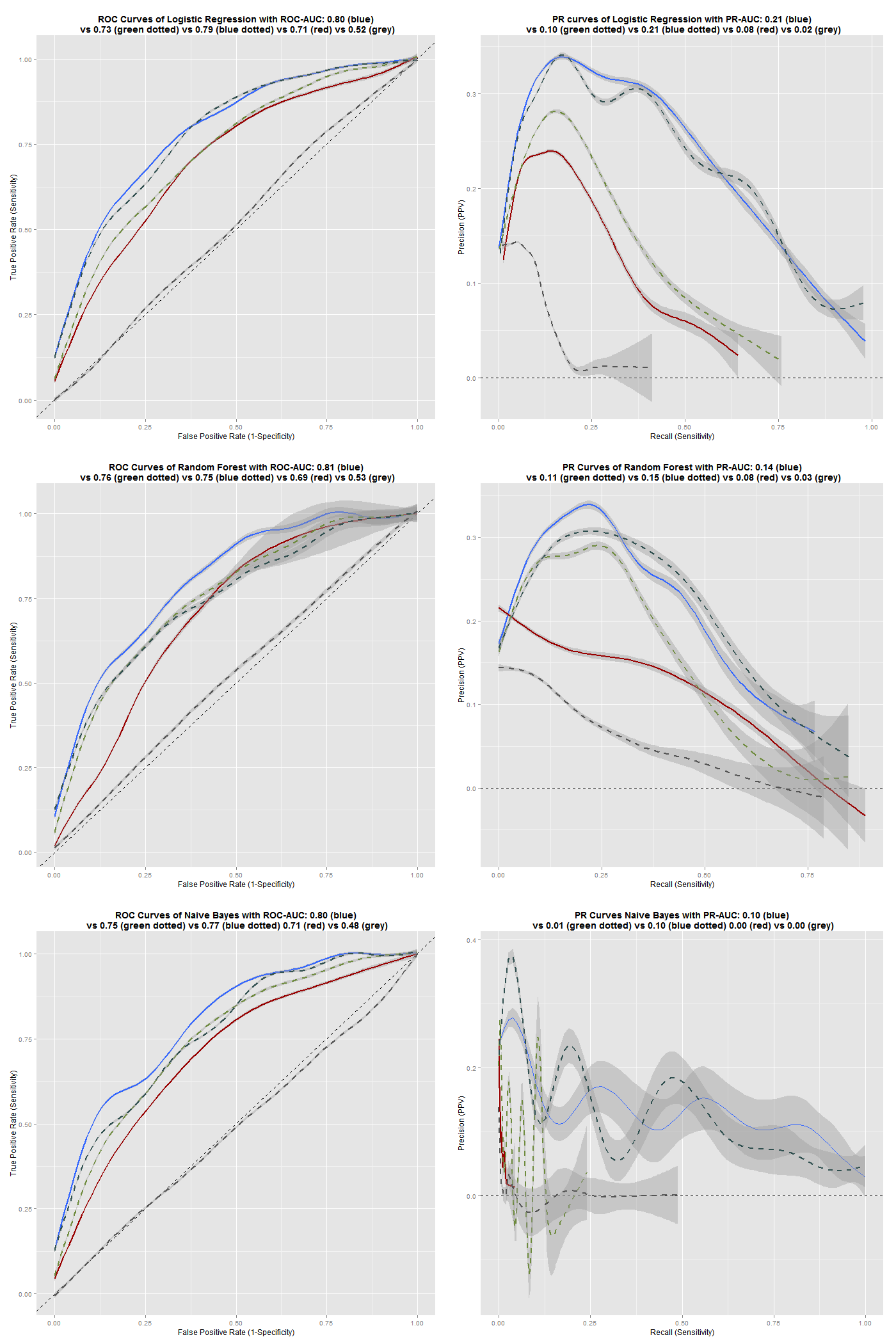


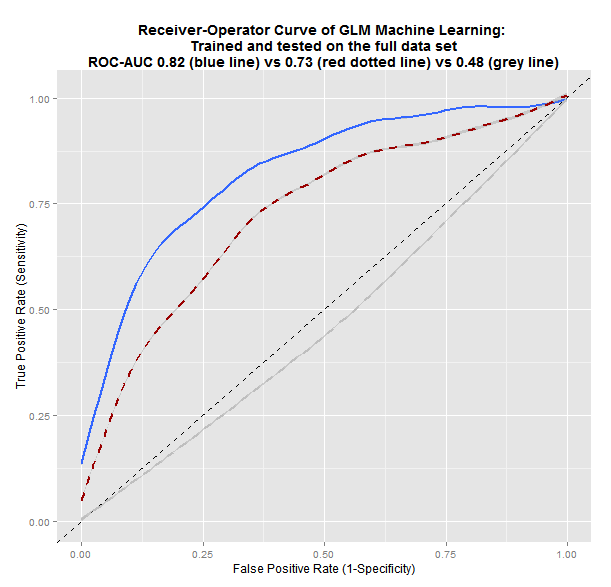
 Prediction model curve using baseline plus impulsivity and compulsivity variables


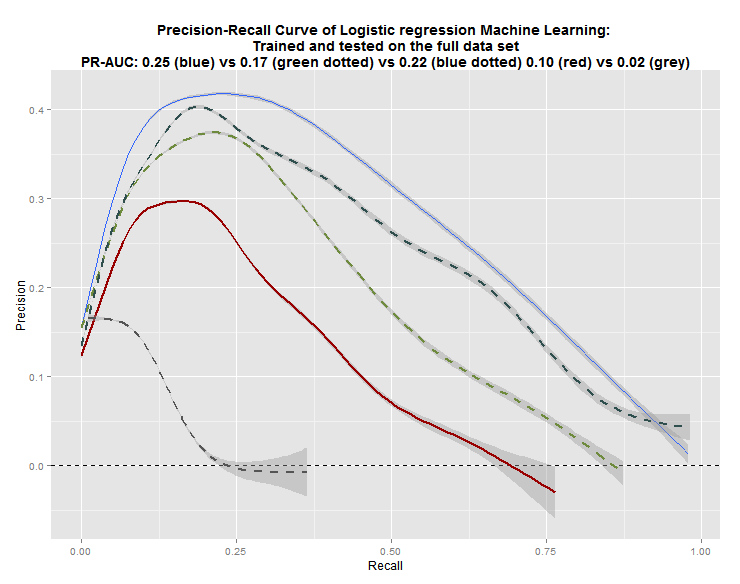
 Prediction model curve using baseline plus impulsivity variables


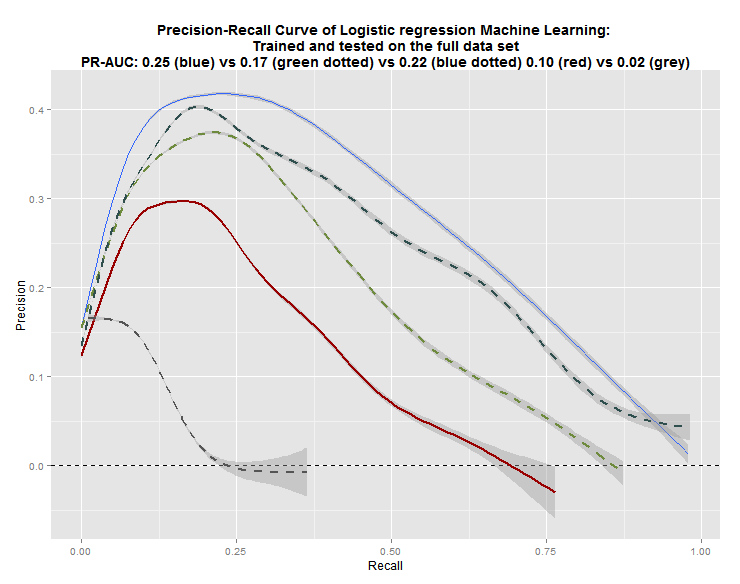
 Prediction model curve using baseline plus compulsivity variables


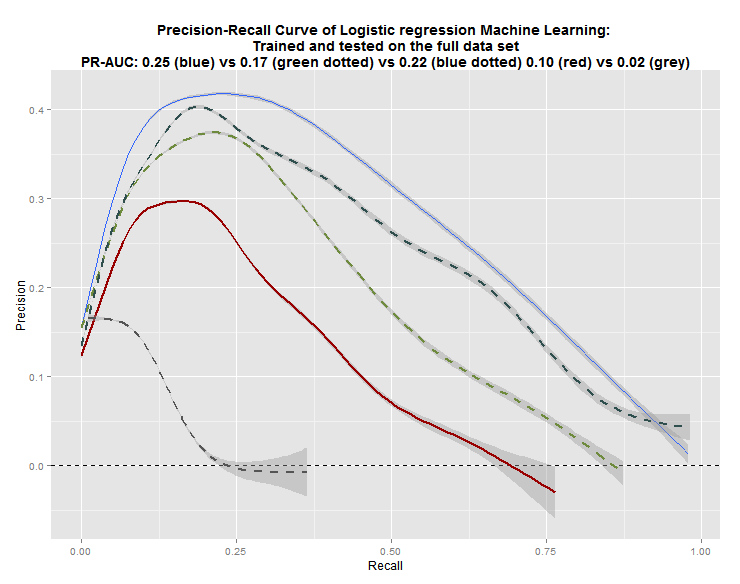
 Prediction model curve using baseline variables only


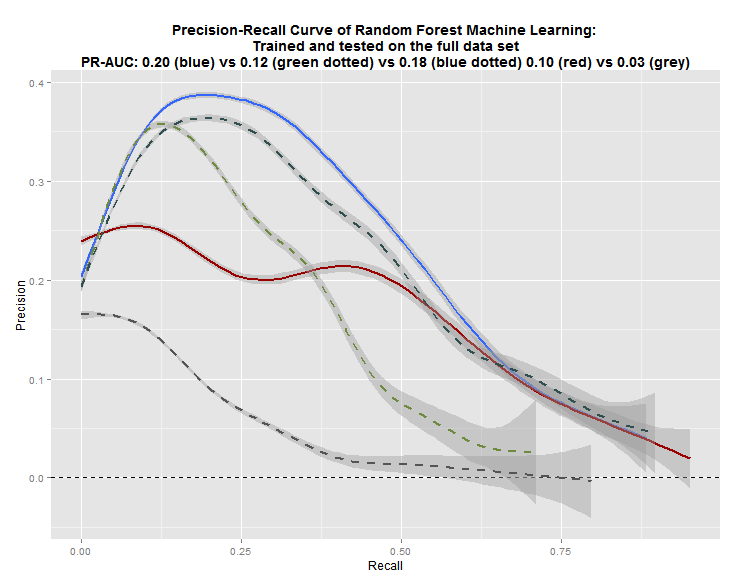
 Prediction model curve ‘at chance’ level with randomized variable scores

# eTable 12: Validation set-up (C) Chicago set basic metrics for all models

| **Model** | **Metrics** | **IMP-COMP set mean ± SD** | **IMP only set mean ± SD** | **COMP only set mean ± SD** | **Baseline set mean ± SD** | **‘Chance level’ set** |
| --- | --- | --- | --- | --- | --- | --- |
| **Logistic Regression** | **ROC-AUC** | 0.85 ± 0.039 | 0.85 ± 0.037 | 0.80 ± 0.051 | 0.74 ± 0.063 | 0.47 ± 0.056 |
|  | **PR-AUC** | 0.36 ± 0.059 | 0.33 ± 0.054 | 0.29 ± 0.049 | 0.15 ± 0.030 | 0.03 ± 0.005 |
|  | **Accuracy** | 0.89 ± 0.021 | 0.89 ± 0.017 | 0.89 ± 0.020 | 0.88 ± 0.012 | 0.88 ± 0.001 |
|  | **Sensitivity** | 0.59 ± 0.145 | 0.58 ± 0.143 | 0.57 ± 0.169 | 0.38 ± 0.337 | 0.00 ± 0.000 |
|  | **Specificity** | 0.92 ± 0.012 | 0.91 ± 0.011 | 0.91 ± 0.011 | 0.90 ± 0.005 | 0.88 ± 0.000 |
|  | **PPV** | 0.33 ± 0.105 | 0.31 ± 0.096 | 0.29 ± 0.093 | 0.06 ± 0.048 | 0.00 ± 0.000 |
|  | **NPV** | 0.97 ± 0.019 | 0.97 ± 0.017 | 0.97 ± 0.019 | 0.99 ± 0.015 | 1.00 ± 0.001 |
|  | **Kappa** | 0.89 ± 0.021 | 0.89 ± 0.018 | 0.89 ± 0.020 | 0.88 ± 0.012 | 0.88 ± 0.001 |
|  | **F Measure** | 0.48 ± 0.111 | 0.46 ± 0.107 | 0.43 ± 0.110 | 0.10 ± 0.083 | 0.00 ± 0.000 |
| **Random Forests** | **ROC-AUC** | 0.89 ± 0.033 | 0.86 ± 0.036 | 0.79 ± 0.034 | 0.69 ± 0.035 | 0.50 ± 0.052 |
|  | **PR-AUC** | 0.29 ± 0.044 | 0.22 ± 0.026 | 0.18 ± 0.030 | 0.10 ± 0.052 | 0.06 ± 0.023 |
|  | **Accuracy** | 0.90 ± 0.012 | 0.89 ± 0.009 | 0.91 ± 0.004 | 0.91 ± 0.009 | 0.88 ± 0.001 |
|  | **Sensitivity** | 0.70 ± 0.201 | 0.68 ± 0.226 | 0.72 ± 0.213 | 0.38 ± 0.175 | 0.00 ± 0.000 |
|  | **Specificity** | 0.91 ± 0.014 | 0.90 ± 0.006 | 0.92 ± 0.004 | 0.91 ± 0.005 | 0.88 ± 0.002 |
|  | **PPV** | 0.24 ± 0.126 | 0.12 ± 0.056 | 0.10 ± 0.053 | 0.06 ± 0.070 | 0.01 ± 0.026 |
|  | **NPV** | 0.99 ± 0.012 | 0.99 ± 0.010 | 0.99 ± 0.005 | 0.99 ± 0.014 | 0.99 ± 0.011 |
|  | **Kappa** | 0.90 ± 0.012 | 0.89 ± 0.009 | 0.91 ± 0.004 | 0.91 ± 0.009 | 0.88 ± 0.009 |
|  | **F Measure** | 0.37 ± 0.152 | 0.20 ± 0.088 | 0.18 ± 0.084 | 0.10 ± 0.120 | 0.02 ± 0.049 |
| **Naïve Bayes** | **ROC-AUC** | 0.87 ± 0.039 | 0.86 ± 0.036 | 0.82 ± 0.047 | 0.75 ± 0.052 | 0.55 ± 0.047 |
|  | **PR-AUC** | 0.49 ± 0.089 | 0.27 ± 0.068 | 0.38 ± 0.078 | 0.01 ± 0.002 | 0.01 ± 0.006 |
|  | **Accuracy** | 0.91 ± 0.021 | 0.89 ± 0.010 | 0.90 ± 0.019 | 0.88 ± 0.000 | 0.88 ± 0.000 |
|  | **Sensitivity** | 0.67 ± 0.149 | 0.69 ± 0.204 | 0.61 ± 0.171 | 0.00 ± 0.000 | 0.00 ± 0.000 |
|  | **Specificity** | 0.93 ± 0.012 | 0.90 ± 0.007 | 0.92 ± 0.011 | 0.88 ± 0.000 | 0.88 ± 0.000 |
|  | **PPV** | 0.44 ± 0.102 | 0.18 ± 0.073 | 0.31 ± 0.092 | 0.00 ± 0.000 | 0.00 ± 0.000 |
|  | **NPV** | 0.97 ± 0.017 | 0.99 ± 0.010 | 0.97 ± 0.016 | 1.00 ± 0.000 | 1.00 ± 0.000 |
|  | **Kappa** | 0.91 ± 0.021 | 0.89 ± 0.010 | 0.89 ± 0.019 | 0.88 ± 0.000 | 0.88 ± 0.000 |
|  | **F Measure** | 0.59 ± 0.096 | 0.29 ± 0.101 | 0.46 ± 0.102 | 0.00 ± 0.000 | 0.00 ± 0.000 |

**AUC** – Area under the curve; **COMP only** – Prediction model using baseline plus compulsivity variables; **F Measure** – balanced F-score (*F_1_*); **IMP only** – Prediction model using baseline plus impulsivity variables; **IMP-COMP** – Prediction model using baseline plus impulsivity-compulsivity variables; **Kappa** – Cohen's kappa coefficient; **NPV** – Negative predictive value; **PPV** – Positive predictive value; **PR** – Precision-Recall (PRC – Precision-Recall Curve); **ROC** – Receiver Operating Characteristic curve; **SD** – Standard deviation

# eTable 13: Validation set-up (C) Chicago set Variable Importance matrices from prediction using baseline plus impulsivity-compulsivity variables of Logistic Regression and Random Forests

|  | **Logistic Regression** | | **Random Forest** | |
| --- | --- | --- | --- | --- |
| **Rank** | **Variable** | **VI %** | **Variable** | **VI %** |
| 1 | BISMI | 97.0 | PI-CC | 100.0 |
| 2 | ASRS | 66.0 | ASRS | 80.0 |
| 3 | Race (non-Caucasian) | 59.0 | BISMI | 74.0 |
| 4 | PI-CC | 41.0 | PI-THSO | 70.0 |
| 5 | Social Anxiety Diagnosis | 37.0 | PI-COWC | 67.0 |
| 6 | PI-COWC | 35.0 | BISAI | 66.0 |
| 7 | BISAI | 32.0 | BISNI | 60.0 |
| 8 | OCD DIAGNOSIS | 26.0 | PI-IHSO | 53.0 |
| 9 | PI-IHSO | 25.0 | Age (older) | 46.0 |
| 10 | GAD DIAGNOSIS | 23.0 | PI-DGC | 43.0 |
| 11 | Gender (Male) | 17.0 | ADHD DIAGNOSIS | 22.0 |
| 12 | Age (older) | 15.0 | OCD DIAGNOSIS | 19.0 |
| 13 | PI-THSO | 15.0 | GAD DIAGNOSIS | 19.0 |
| 14 | ADHD DIAGNOSIS | 14.0 | Social Anxiety Diagnosis | 18.0 |
| 15 | BISNI | 14.0 | Race (non-Caucasian) | 17.0 |
| 16 | PI-DGC | 14.0 | Education (Some College) | 12.0 |
| 17 | Education (High School) | 13.0 | Gender (Male) | 10.0 |
| 18 | Education (Some College) | 10.0 | Education (College) | 9.4 |
| 19 | Education (College) | 9.8 | Education (High School) | 6.6 |
| 20 | Education (Beyond College) | 7.6 | Education (Beyond College) | 5.9 |

**ADHD –** Attention Deficit Hyperactivity Disorder**, ASRS** - Adult ADHD Self-Report Scale (ASRS-v1.1), **BISAI** - Barratt Impulsiveness Scale 11 Attention Impulsivity factor (BIS), **BISMI** - Barratt Impulsiveness Scale 11 Motor Impulsivity factor (BIS), **BISNI** - Barratt Impulsiveness Scale 11 Non-planning Impulsivity factor (BIS), **GAD** – Generalized Anxiety disorder, **OCD** – Obsessive-Compulsive disorder, **PI-CC** – Padua Inventory-Revised Checking Compulsion, **PI-COWC** – Padua Inventory-Revised Contamination Obsessions and Washing Compulsions, **PI-DGC** – Padua Inventory-Revised Dressing and Grooming Compulsions, **PI-IHSO** – Padua Inventory-Revised Impulses to Harm Self or Others, **PI-THSO** – Padua Inventory-Revised Thoughts of Harm to Self or Others

# eFigure 3: Validation set-up (C) Chicago set Receiver Operating Characteristic and Precision-Recall Curves for all models


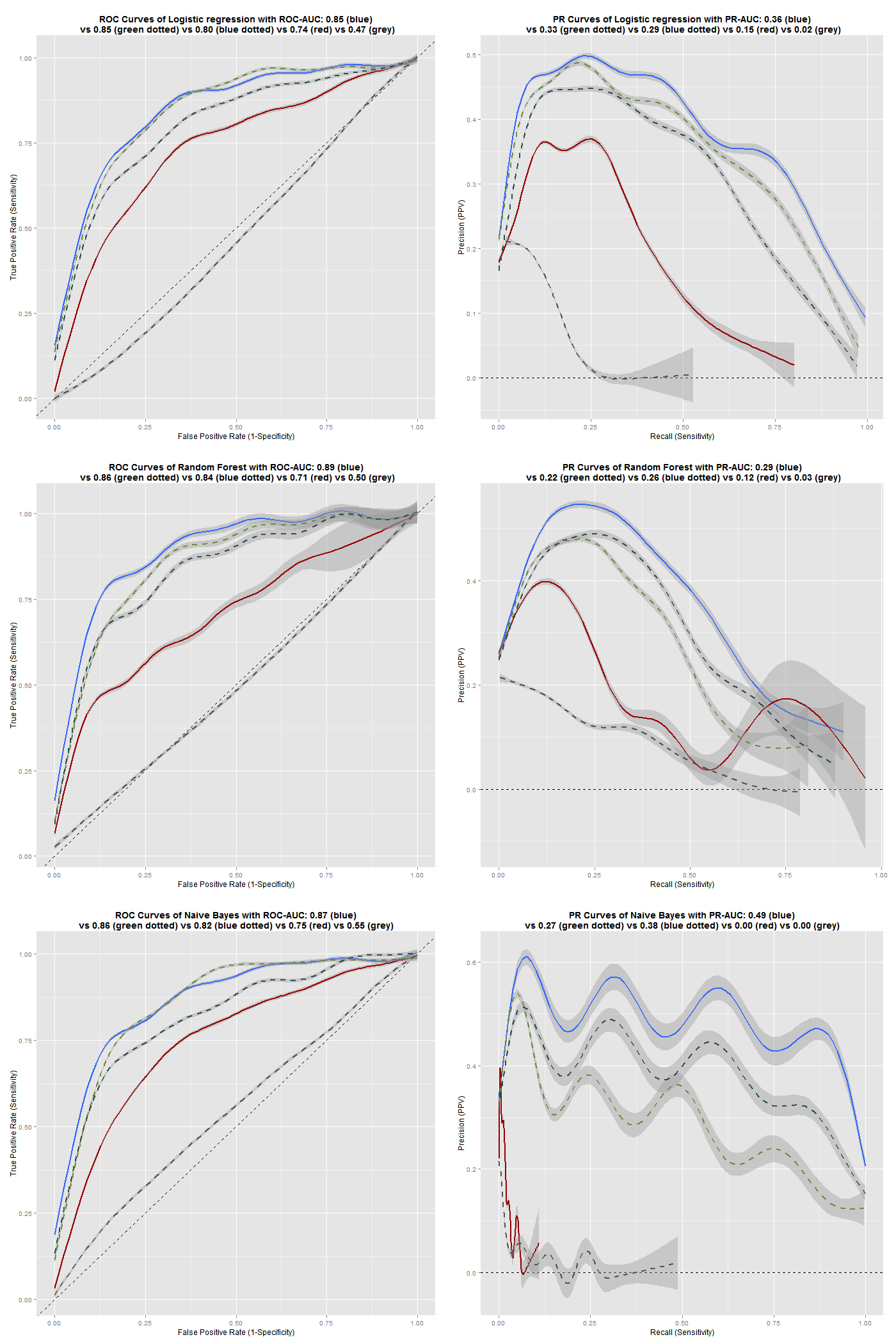


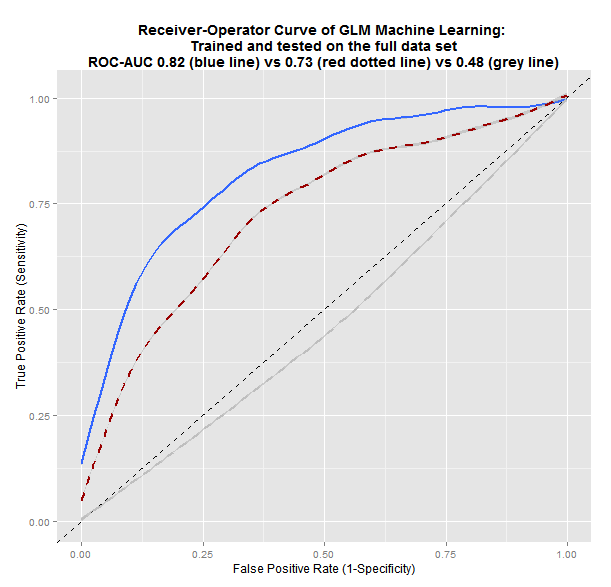
 Prediction model curve using baseline plus impulsivity and compulsivity variables


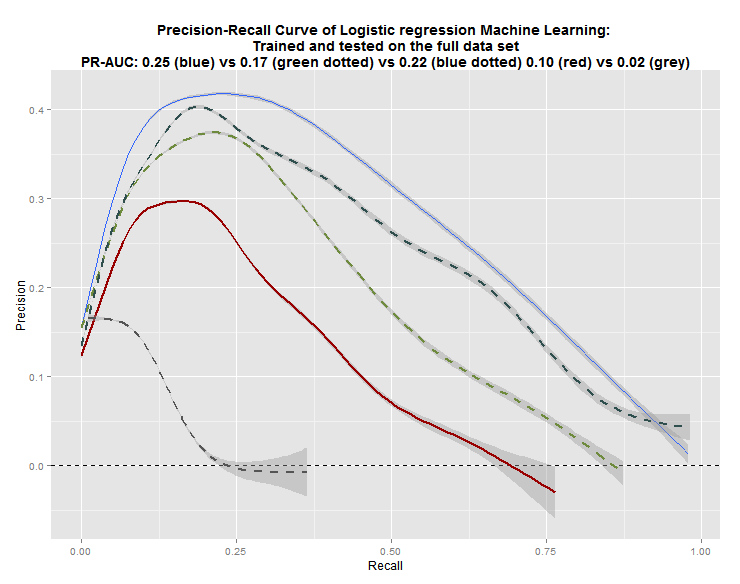
 Prediction model curve using baseline plus impulsivity variables


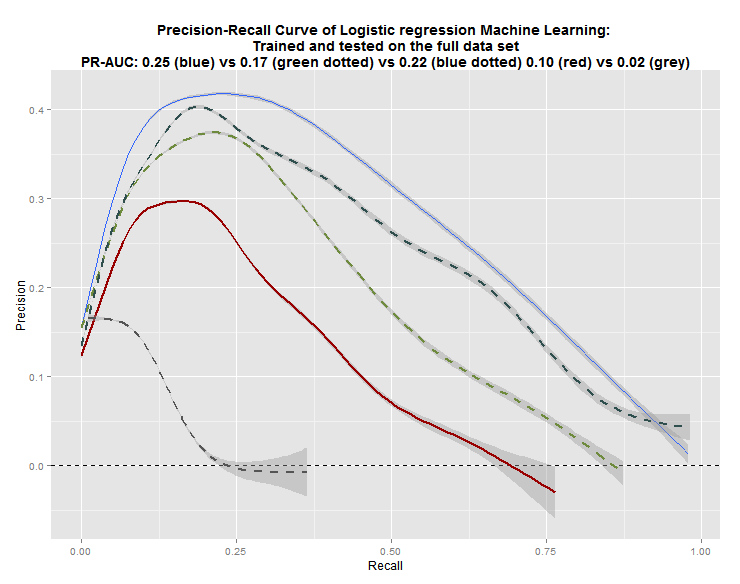
 Prediction model curve using baseline plus compulsivity variables


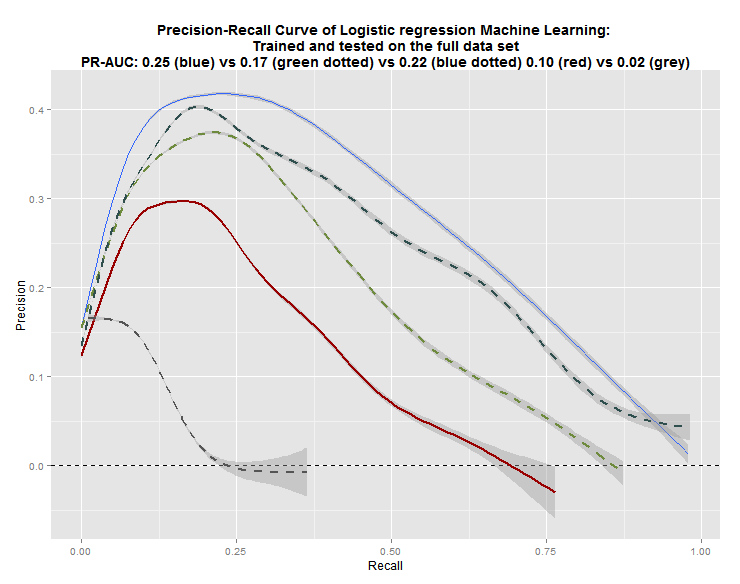
 Prediction model curve using baseline variables only


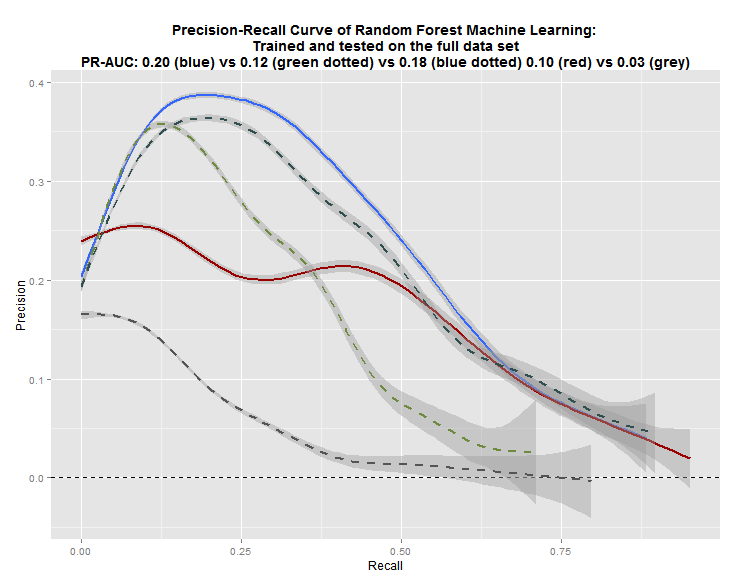
 Prediction model curve ‘at chance’ level with randomized variable scores

# eTable 14: Validation set-up (D) trained on the Stellenbosch set and tested on the Chicago set basic metrics for all models

| **Model** | **Metrics** | **IMP-COMP set mean ± SD** | **IMP only set mean ± SD** | **COMP only set mean ± SD** | **Baseline set mean ± SD** | **‘Chance level’ set** |
| --- | --- | --- | --- | --- | --- | --- |
| **Logistic Regression** | **ROC-AUC** | 0.84 ± 0.001 | 0.82 ± 0.011 | 0.78 ± 0.006 | 0.71 ± 0.006 | 0.52 ± 0.009 |
|  | **PR-AUC** | 0.28 ± 0.011 | 0.18 ± 0.009 | 0.23 ± 0.010 | 0.12 ± 0.006 | 0.03 ± 0.002 |
|  | **Accuracy** | 0.89 ± 0.002 | 0.88 ± 0.002 | 0.89 ± 0.002 | 0.88 ± 0.001 | 0.88 ± 0.000 |
|  | **Sensitivity** | 0.63 ± 0.031 | 0.71 ± 0.051 | 0.56 ± 0.027 | 0.35 ± 0.109 | 0.00 ± 0.000 |
|  | **Specificity** | 0.90 ± 0.001 | 0.89 ± 0.002 | 0.90 ± 0.002 | 0.88 ± 0.001 | 0.88 ± 0.000 |
|  | **PPV** | 0.21 ± 0.013 | 0.08 ± 0.018 | 0.17 ± 0.015 | 0.02 ± 0.013 | 0.00 ± 0.000 |
|  | **NPV** | 0.98 ± 0.002 | 0.99 ± 0.001 | 0.98 ± 0.002 | 0.99 ± 0.001 | 1.00 ± 0.000 |
|  | **Kappa** | 0.89 ± 0.002 | 0.89 ± 0.002 | 0.89 ± 0.002 | 0.88 ± 0.001 | 0.88 ± 0.000 |
|  | **F Measure** | 0.35 ± 0.017 | 0.15 ± 0.029 | 0.29 ± 0.021 | 0.04 ± 0.025 | 0.00 ± 0.000 |
| **Random Forests** | **ROC-AUC** | 0.84 ± 0.001 | 0.73 ± 0.026 | 0.79 ± 0.009 | 0.67 ± 0.011 | 0.51 ± 0.014 |
|  | **PR-AUC** | 0.22 ± 0.030 | 0.13 ± 0.021 | 0.21 ± 0.024 | 0.14 ± 0.020 | 0.04 ± 0.010 |
|  | **Accuracy** | 0.89 ± 0.006 | 0.88 ± 0.007 | 0.89 ± 0.004 | 0.86 ± 0.009 | 0.88 ± 0.002 |
|  | **Sensitivity** | 0.68 ± 0.122 | 0.27 ± 0.160 | 0.64 ± 0.153 | 0.29 ± 0.044 | 0.00 ± 0.000 |
|  | **Specificity** | 0.90 ± 0.007 | 0.89 ± 0.004 | 0.90 ± 0.003 | 0.89 ± 0.003 | 0.88 ± 0.001 |
|  | **PPV** | 0.16 ± 0.072 | 0.06 ± 0.045 | 0.14 ± 0.036 | 0.11 ± 0.036 | 0.00 ± 0.005 |
|  | **NPV** | 0.99 ± 0.004 | 0.99 ± 0.013 | 0.99 ± 0.007 | 0.96 ± 0.014 | 0.99 ± 0.003 |
|  | **Kappa** | 0.89 ± 0.005 | 0.88 ± 0.007 | 0.89 ± 0.004 | 0.86 ± 0.009 | 0.88 ± 0.002 |
|  | **F Measure** | 0.27 ± 0.115 | 0.10 ± 0.080 | 0.23 ± 0.057 | 0.19 ± 0.060 | 0.00 ± 0.009 |
| **Naïve Bayes** | **ROC-AUC** | 0.86 ± 0.002 | 0.86 ± 0.003 | 0.84 ± 0.002 | 0.71 ± 0.008 | 0.51 ± 0.012 |
|  | **PR-AUC** | 0.15 ± 0.011 | 0.01 ± 0.002 | 0.10 ± 0.010 | 0.00 ± 0.001 | 0.00 ± 0.000 |
|  | **Accuracy** | 0.89 ± 0.002 | 0.88 ± 0.000 | 0.89 ± 0.001 | 0.88 ± 0.000 | 0.88 ± 0.000 |
|  | **Sensitivity** | 0.75 ± 0.072 | 0.00 ± 0.000 | 0.90 ± 0.136 | 0.00 ± 0.000 | 0.00 ± 0.000 |
|  | **Specificity** | 0.89 ± 0.001 | 0.88 ± 0.000 | 0.89 ± 0.001 | 0.88 ± 0.000 | 0.88 ± 0.000 |
|  | **PPV** | 0.08 ± 0.011 | 0.00 ± 0.000 | 0.04 ± 0.006 | 0.00 ± 0.000 | 0.00 ± 0.000 |
|  | **NPV** | 0.99 ± 0.001 | 1.00 ± 0.000 | 0.99 ± 0.001 | 1.00 ± 0.000 | 1.00 ± 0.000 |
|  | **Kappa** | 0.89 ± 0.002 | 0.99 ± 0.000 | 0.89 ± 0.001 | 0.88 ± 0.000 | 0.88 ± 0.000 |
|  | **F Measure** | 0.14 ± 0.019 | 0.00 ± 0.000 | 0.07 ± 0.011 | 0.00 ± 0.000 | 0.00 ± 0.000 |

**AUC** – Area under the curve; **COMP only** – Prediction model using baseline plus compulsivity variables; **F Measure** – balanced F-score (*F_1_*); **IMP only** – Prediction model using baseline plus impulsivity variables; **IMP-COMP** – Prediction model using baseline plus impulsivity-compulsivity variables; **Kappa** – Cohen's kappa coefficient; **NPV** – Negative predictive value; **PPV** – Positive predictive value; **PR** – Precision-Recall (PRC – Precision-Recall Curve); **ROC** – Receiver Operating Characteristic curve; **SD** – Standard deviation

# eTable 15: Validation set-up (D) trained on the Stellenbosch set and tested on the Chicago set Variable Importance matrices from prediction using baseline plus impulsivity-compulsivity variables of Logistic Regression and Random Forests

|  | **Logistic Regression** | | **Random Forest** | |
| --- | --- | --- | --- | --- |
| **Rank** | **Variable** | **VI %** | **Variable** | **VI %** |
| 1 | Age (older) | 100.0 | PI-IHSO | 98.0 |
| 2 | PI-IHSO | 75.0 | Age (older) | 93.0 |
| 3 | Race (non-Caucasian) | 64.0 | ASRS | 89.0 |
| 4 | PI-CC | 49.000 | PI-CC | 81.0 |
| 5 | PI-DGC | 39.000 | BISMI | 66.0 |
| 6 | ADHD DIAGNOSIS | 26.000 | PI-COWC | 66.0 |
| 7 | BISMI | 26.0 | PI-THSO | 66.0 |
| 8 | PI-COWC | 24.0 | BISNI | 65.0 |
| 9 | PI-THSO | 23.0 | PI-DGC | 59.0 |
| 10 | BISNI | 19.0 | BISAI | 59.0 |
| 11 | GAD DIAGNOSIS | 9.6 | Race (non-Caucasian) | 19.0 |
| 12 | ASRS | 9.2 | Education (Some College) | 12.0 |
| 13 | BISAI | 7.3 | ADHD DIAGNOSIS | 11.0 |
| 14 | Social Anxiety Diagnosis | 6.2 | Gender (Male) | 11.0 |
| 15 | Gender (Male) | 5.4 | OCD DIAGNOSIS | 10.0 |
| 16 | OCD DIAGNOSIS | 4.6 | GAD DIAGNOSIS | 10.0 |
| 17 | Education (Some College) | 3.0 | Social Anxiety Diagnosis | 9.4 |
| 18 | Education (High School) | 2.6 | Education (College) | 9.3 |
| 19 | Education (College) | 1.8 | Education (High School) | 8.4 |
| 20 | Education (Beyond College) | 0.03 | Education (Beyond College) | 7.3 |

**ADHD –** Attention Deficit Hyperactivity Disorder**, ASRS** - Adult ADHD Self-Report Scale (ASRS-v1.1), **BISAI** - Barratt Impulsiveness Scale 11 Attention Impulsivity factor (BIS), **BISMI** - Barratt Impulsiveness Scale 11 Motor Impulsivity factor (BIS), **BISNI** - Barratt Impulsiveness Scale 11 Non-planning Impulsivity factor (BIS), **GAD** – Generalized Anxiety disorder, **OCD** – Obsessive-Compulsive disorder, **PI-CC** – Padua Inventory-Revised Checking Compulsion, **PI-COWC** – Padua Inventory-Revised Contamination Obsessions and Washing Compulsions, **PI-DGC** – Padua Inventory-Revised Dressing and Grooming Compulsions, **PI-IHSO** – Padua Inventory-Revised Impulses to Harm Self or Others, **PI-THSO** – Padua Inventory-Revised Thoughts of Harm to Self or Others

# eFigure 4: Validation set-up (D) trained on the Stellenbosch set and tested on the Chicago set Receiver Operating Characteristic and Precision-Recall Curves for all models


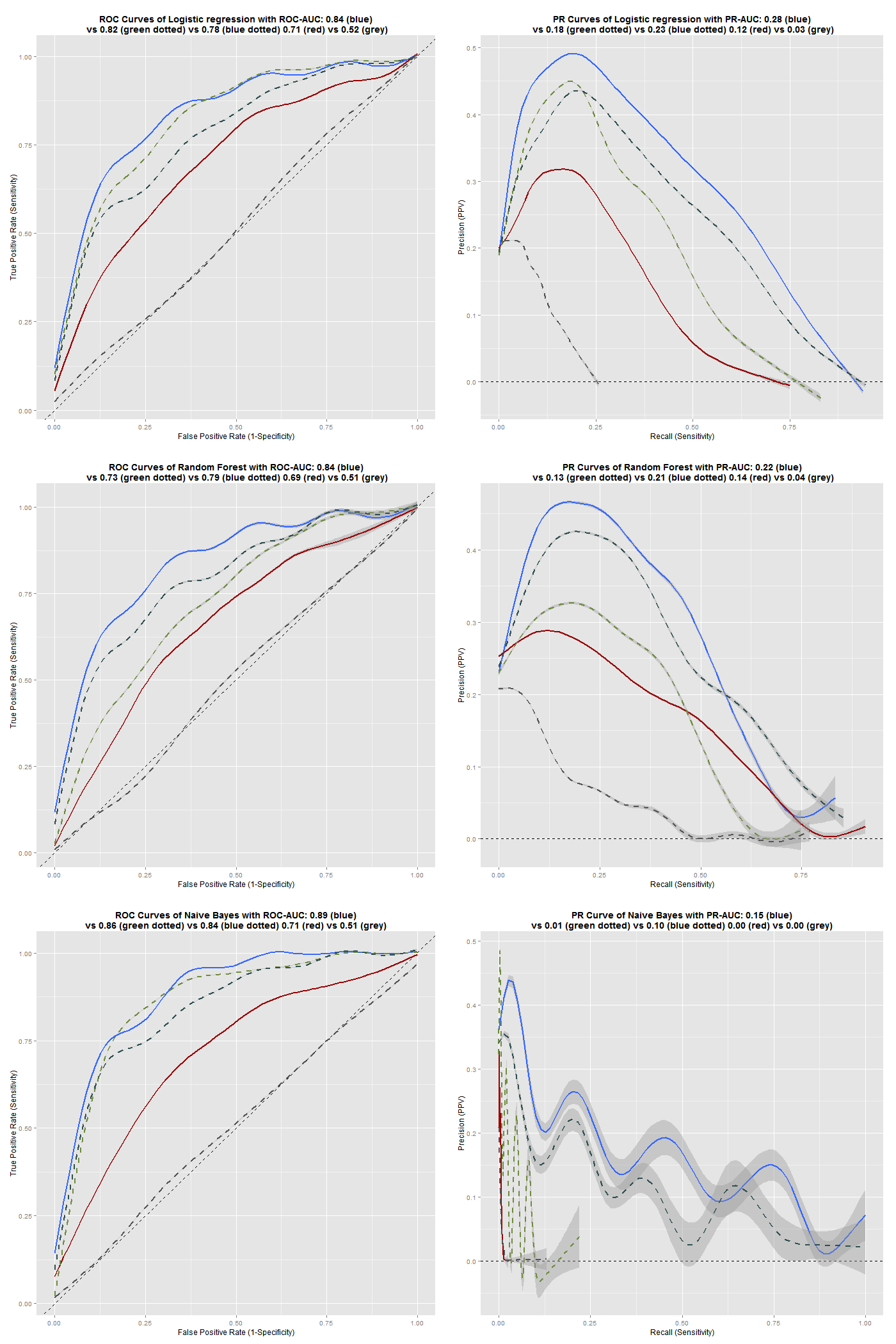


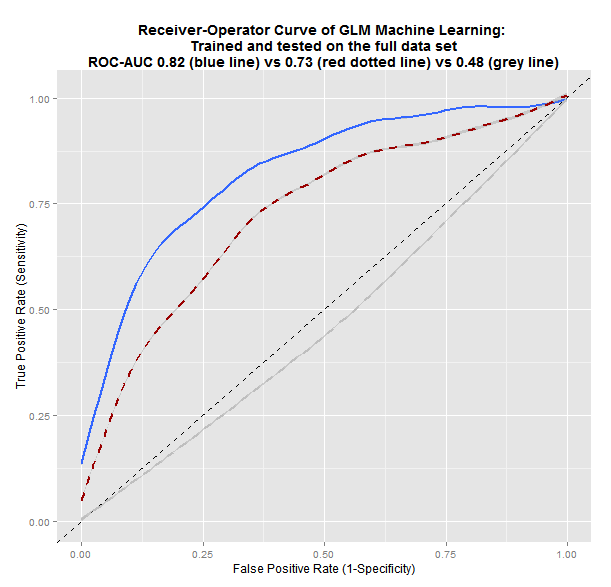
 Prediction model curve using baseline plus impulsivity and compulsivity variables


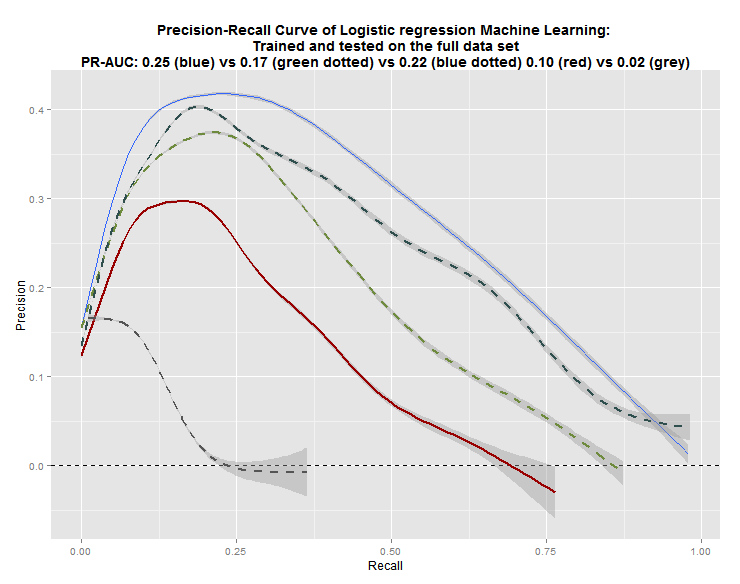
 Prediction model curve using baseline plus impulsivity variables


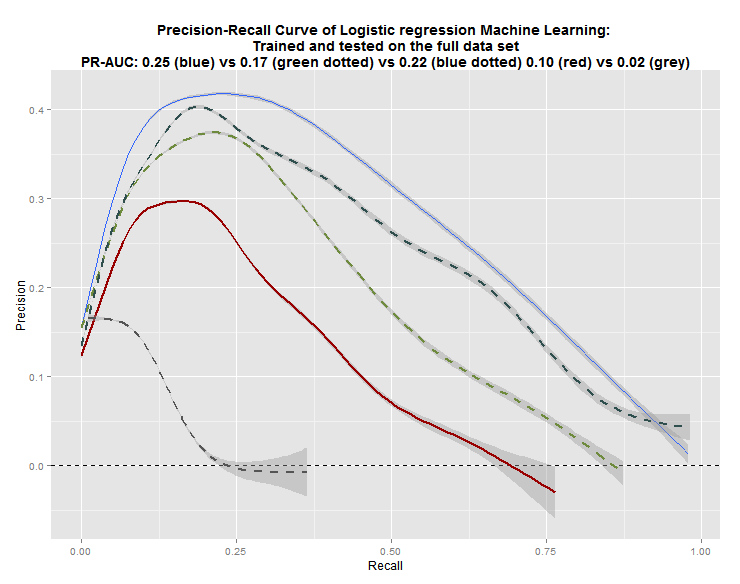
 Prediction model curve using baseline plus compulsivity variables


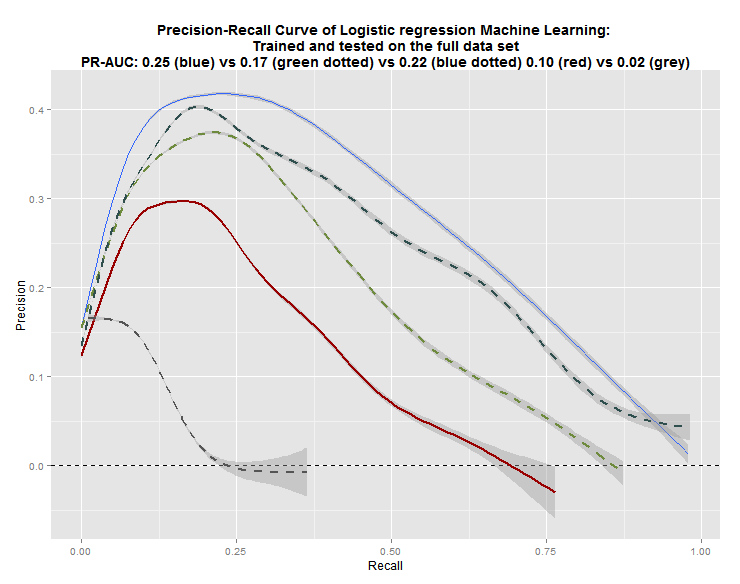
 Prediction model curve using baseline variables only


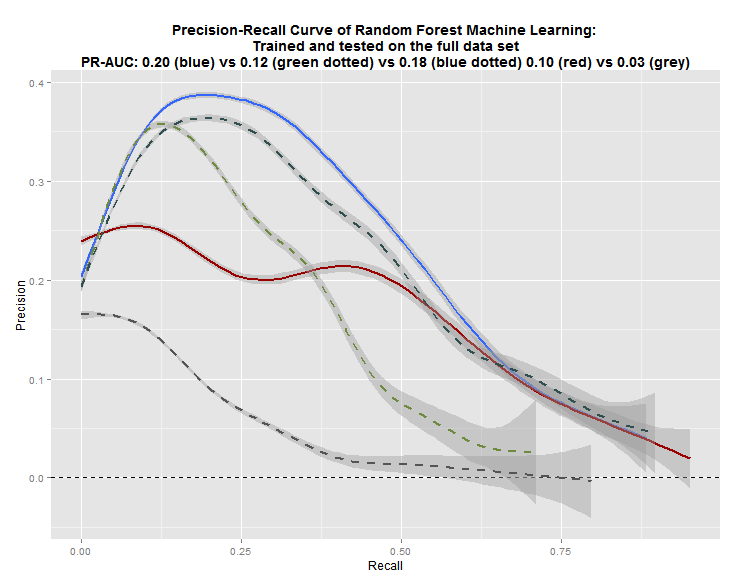
 Prediction model curve ‘at chance’ level with randomized variable scores

# eTable 16: Validation set-up (E) trained on the Chicago set and tested on the Stellenbosch set basic metrics for all models

| **Model** | **Metrics** | **IMP-COMP set mean ± SD** | **IMP only set mean ± SD** | **COMP only set mean ± SD** | **Baseline set mean ± SD** | **‘Chance level’ set** |
| --- | --- | --- | --- | --- | --- | --- |
| **Logistic Regression** | **ROC-AUC** | 0.77 ± 0.009 | 0.73 ± 0.005 | 0.73 ± 0.009 | 0.67 ± 0.014 | 0.54 ± 0.009 |
|  | **PR-AUC** | 0.22 ± 0.007 | 0.18 ± 0.007 | 0.21 ± 0.008 | 0.11 ± 0.007 | 0.03 ± 0.002 |
|  | **Accuracy** | 0.90 ± 0.005 | 0.89 ± 0.006 | 0.91 ± 0.005 | 0.92 ± 0.001 | 0.92 ± 0.000 |
|  | **Sensitivity** | 0.33 ± 0.024 | 0.25 ± 0.020 | 0.35 ± 0.032 | 0.32 ± 0.007 | 0.00 ± 0.000 |
|  | **Specificity** | 0.94 ± 0.002 | 0.94 ± 0.001 | 0.94 ± 0.001 | 0.93 ± 0.001 | 0.92 ± 0.000 |
|  | **PPV** | 0.29 ± 0.024 | 0.24 ± 0.021 | 0.25 ± 0.017 | 0.09 ± 0.005 | 0.00 ± 0.000 |
|  | **NPV** | 0.95 ± 0.006 | 0.94 ± 0.008 | 0.96 ± 0.005 | 0.98 ± 0.001 | 1.00 ± 0.000 |
|  | **Kappa** | 0.90 ± 0.005 | 0.89 ± 0.007 | 0.91 ± 0.005 | 0.92 ± 0.001 | 0.92 ± 0.000 |
|  | **F Measure** | 0.47 ± 0.028 | 0.37 ± 0.027 | 0.39 ± 0.021 | 0.17 ± 0.009 | 0.00 ± 0.000 |
| **Random Forests** | **ROC-AUC** | 0.76 ± 0.006 | 0.72 ± 0.005 | 0.72 ± 0.005 | 0.63 ± 0.015 | 0.51 ± 0.011 |
|  | **PR-AUC** | 0.15 ± 0.008 | 0.11 ± 0.005 | 0.14 ± 0.005 | 0.06 ± 0.013 | 0.04 ± 0.009 |
|  | **Accuracy** | 0.91 ± 0.017 | 0.91 ± 0.003 | 0.91 ± 0.004 | 0.92 ± 0.005 | 0.91 ± 0.010 |
|  | **Sensitivity** | 0.35 ± 0.066 | 0.19 ± 0.063 | 0.31 ± 0.031 | 0.28 ± 0.374 | 0.00 ± 0.000 |
|  | **Specificity** | 0.93 ± 0.002 | 0.92 ± 0.002 | 0.93 ± 0.002 | 0.92 ± 0.002 | 0.92 ± 0.001 |
|  | **PPV** | 0.14 ± 0.049 | 0.05 ± 0.023 | 0.15 ± 0.026 | 0.01 ± 0.029 | 0.01 ± 0.006 |
|  | **NPV** | 0.97 ± 0.022 | 0.98 ± 0.005 | 0.97 ± 0.005 | 0.99 ± 0.008 | 0.99 ± 0.011 |
|  | **Kappa** | 0.91 ± 0.018 | 0.91 ± 0.004 | 0.91 ± 0.004 | 0.92 ± 0.005 | 0.91 ± 0.010 |
|  | **F Measure** | 0.25 ± 0.069 | 0.09 ± 0.041 | 0.25 ± 0.039 | 0.01 ± 0.050 | 0.01 ± 0.011 |
| **Naïve Bayes** | **ROC-AUC** | 0.77 ± 0.003 | 0.72 ± 0.005 | 0.73 ± 0.002 | 0.68 ± 0.006 | 0.49 ± 0.008 |
|  | **PR-AUC** | 0.32 ± 0.005 | 0.15 ± 0.010 | 0.25 ± 0.006 | 0.01 ± 0.001 | 0.01 ± 0.001 |
|  | **Accuracy** | 0.90 ± 0.002 | 0.92 ± 0.002 | 0.91 ± 0.002 | 0.92 ± 0.000 | 0.92 ± 0.001 |
|  | **Sensitivity** | 0.35 ± 0.011 | 0.33 ± 0.033 | 0.35 ± 0.016 | 0.00 ± 0.000 | 0.00 ± 0.000 |
|  | **Specificity** | 0.94 ± 0.001 | 0.93 ± 0.001 | 0.94 ± 0.001 | 0.92 ± 0.000 | 0.92 ± 0.000 |
|  | **PPV** | 0.35 ± 0.014 | 0.11 ± 0.016 | 0.20 ± 0.014 | 0.00 ± 0.000 | 0.00 ± 0.000 |
|  | **NPV** | 0.94 ± 0.004 | 0.98 ± 0.002 | 0.97 ± 0.003 | 1.00 ± 0.000 | 0.99 ± 0.001 |
|  | **Kappa** | 0.90 ± 0.003 | 0.92 ± 0.002 | 0.91 ± 0.002 | 0.92 ± 0.000 | 0.92 ± 0.001 |
|  | **F Measure** | 0.51 ± 0.015 | 0.20 ± 0.025 | 0.33 ± 0.020 | 0.00 ± 0.000 | 0.00 ± 0.000 |

**AUC** – Area under the curve; **COMP only** – Prediction model using baseline plus compulsivity variables; **F Measure** – balanced F-score (*F_1_*); **IMP only** – Prediction model using baseline plus impulsivity variables; **IMP-COMP** – Prediction model using baseline plus impulsivity-compulsivity variables; **Kappa** – Cohen's kappa coefficient; **NPV** – Negative predictive value; **PPV** – Positive predictive value; **PR** – Precision-Recall (PRC – Precision-Recall Curve); **ROC** – Receiver Operating Characteristic curve; **SD** – Standard deviation

# eTable 17: Validation set-up (E) trained on the Chicago set and tested on the Stellenbosch set Variable Importance matrices from prediction using baseline plus impulsivity-compulsivity variables of Logistic Regression and Random Forests

|  | **Logistic Regression** | | **Random Forest** | |
| --- | --- | --- | --- | --- |
| **Rank** | **Variable** | **VI %** | **Variable** | **VI %** |
| 1 | BISMI | 100.0 | PI-CC | 100.0 |
| 2 | ASRS | 66.0 | ASRS | 78.0 |
| 3 | Race (non-Caucasian) | 56.0 | BISMI | 72.0 |
| 4 | PI-CC | 42.0 | PI-THSO | 68.0 |
| 5 | BISAI | 38.0 | BISAI | 66.0 |
| 6 | PI-COWC | 38.0 | PI-COWC | 65.0 |
| 7 | Social Anxiety Diagnosis | 34.0 | BISNI | 59.0 |
| 8 | OCD DIAGNOSIS | 29.0 | PI-IHSO | 52.0 |
| 9 | PI-IHSO | 21.0 | Age (older) | 45.0 |
| 10 | Education (High School) | 21.0 | PI-DGC | 43.0 |
| 11 | Education (Some College) | 18.0 | ADHD DIAGNOSIS | 22.0 |
| 12 | Education (College) | 17.0 | GAD DIAGNOSIS | 20.0 |
| 13 | GAD DIAGNOSIS | 16.0 | OCD DIAGNOSIS | 19.0 |
| 14 | PI-DGC | 13.0 | Social Anxiety Diagnosis | 18.0 |
| 15 | Age (older) | 12.0 | Race (non-Caucasian) | 17.0 |
| 16 | BISNI | 11.0 | Education (Some College) | 12.0 |
| 17 | ADHD DIAGNOSIS | 10.0 | Gender (Male) | 9.7 |
| 18 | PI-THSO | 10.0 | Education (College) | 8.9 |
| 19 | Gender (Male) | 7.9 | Education (High School) | 6.6 |
| 20 | Education (Beyond College) | 7.8 | Education (Beyond College) | 5.8 |

**ADHD –** Attention Deficit Hyperactivity Disorder**, ASRS** - Adult ADHD Self-Report Scale (ASRS-v1.1), **BISAI** - Barratt Impulsiveness Scale 11 Attention Impulsivity factor (BIS), **BISMI** - Barratt Impulsiveness Scale 11 Motor Impulsivity factor (BIS), **BISNI** - Barratt Impulsiveness Scale 11 Non-planning Impulsivity factor (BIS), **GAD** – Generalized Anxiety disorder, **OCD** – Obsessive-Compulsive disorder, **PI-CC** – Padua Inventory-Revised Checking Compulsion, **PI-COWC** – Padua Inventory-Revised Contamination Obsessions and Washing Compulsions, **PI-DGC** – Padua Inventory-Revised Dressing and Grooming Compulsions, **PI-IHSO** – Padua Inventory-Revised Impulses to Harm Self or Others, **PI-THSO** – Padua Inventory-Revised Thoughts of Harm to Self or Others

# eFigure 5: Validation set-up (E) trained on the Chicago set and tested on the Stellenbosch set Receiver Operating Characteristic and Precision-Recall Curves for all models


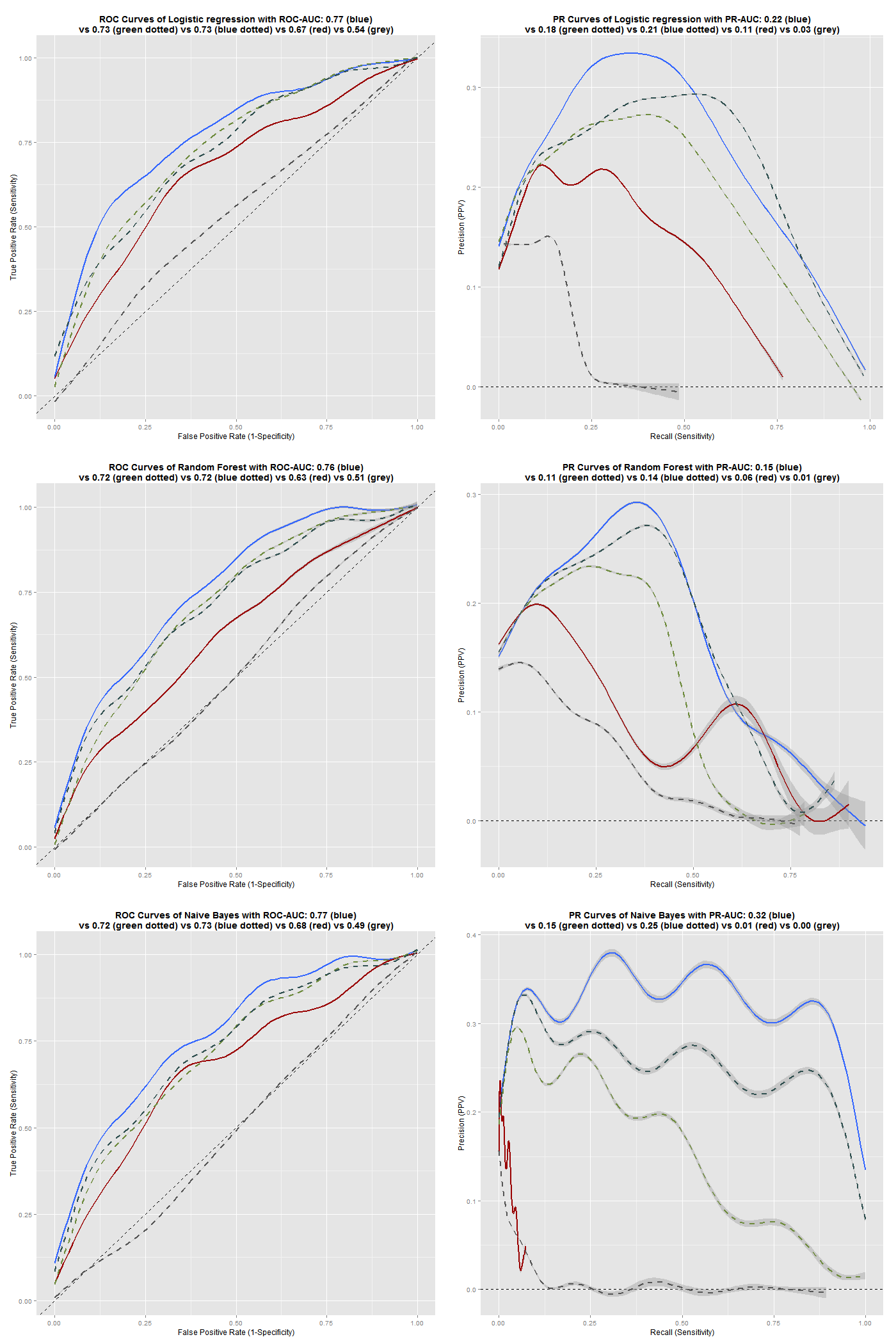


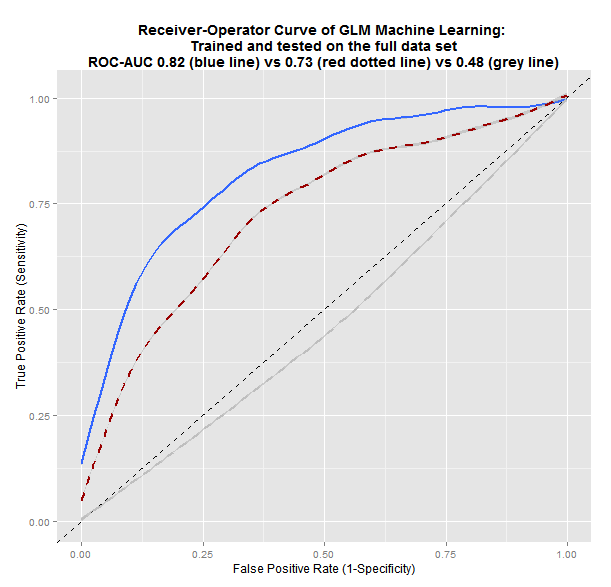
 Prediction model curve using baseline plus impulsivity and compulsivity variables


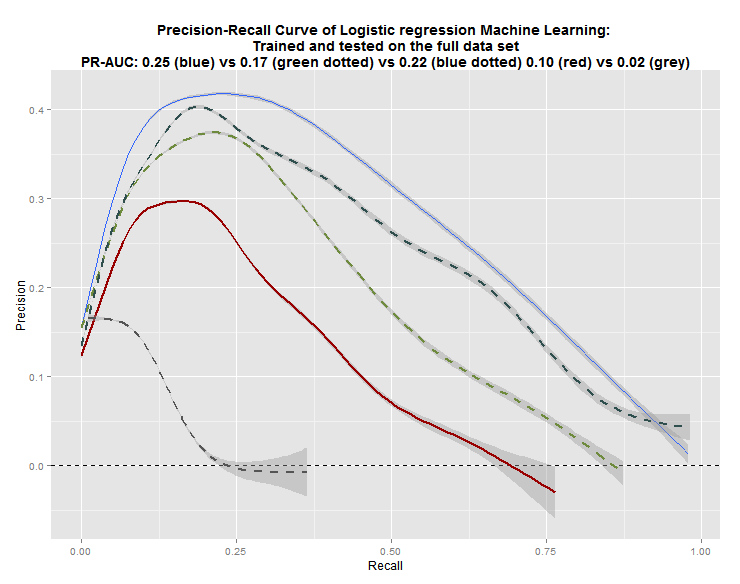
 Prediction model curve using baseline plus impulsivity variables


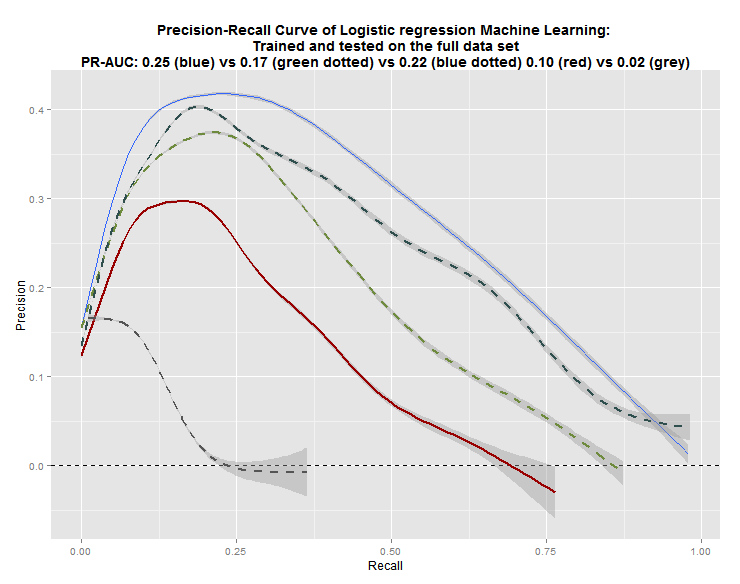
 Prediction model curve using baseline plus compulsivity variables


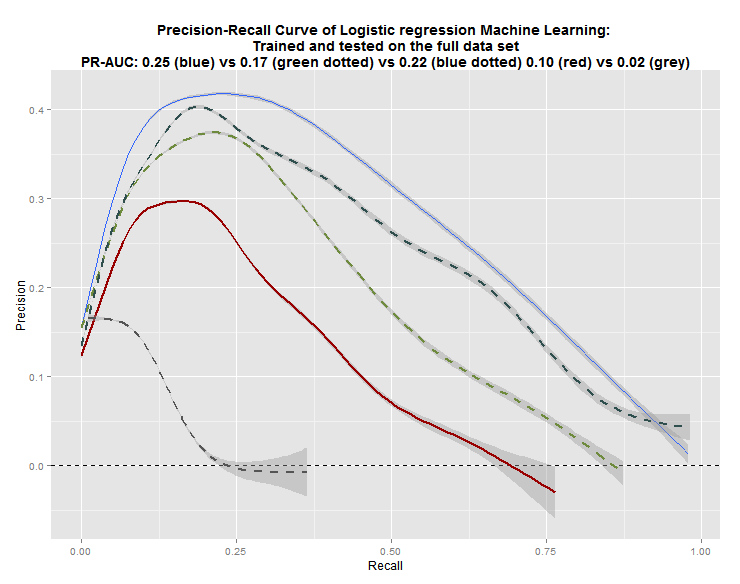
 Prediction model curve using baseline variables only


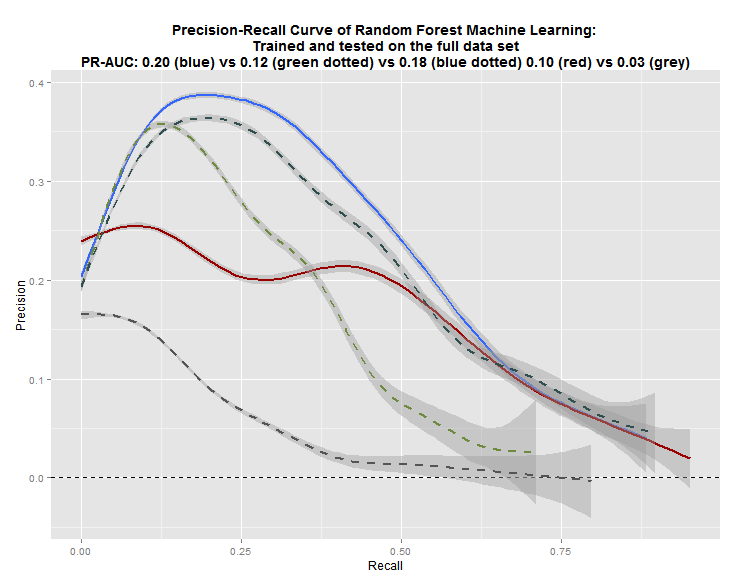
 Prediction model curve ‘at chance’ level with randomized variable scores

# R code for the analysis (example)

#This R code here was used to perform the analysis of the Problematic internet use (PIU): associations with

#the impulsive-compulsive spectrum - An application of machine learning in psychiatry

#parts of the code are missing as this is a draft (submitted for publication) version of the paper.

#reading the data into R space

df <- read.csv("~/Psychiatry Research/ICOCS/df.csv", head = TRUE, stringsAsFactors = FALSE); df <- data.table(df)

#standardizing variables apart from age to allow age for anova analysis later on

df2 <- df %>%

mutate(ASRS = ASRS1 + ASRS2 +ASRS3 + ASRS4 +ASRS5 + ASRS6) %>%

mutate_each_(funs(scale), vars = c("ASRS", "BISAI", "BISMI", "BISNI",

"PADUA.Contamination.Obsessions.and.Washing.Compulsions", "PADUA.Dressing.Grooming.Compulsions",

"PADUA.Checking.Compulsion" , "PADUA.Thoughts.of.Harm.to.Self.Others", "PADUA.Impulses.to.Harm.Self.Others",

"PADUATotal"))

#selecting variables of importance for the analysis

df3 <- df2 %>%

dplyr::select(IAT.Class, IAT.Total, BIS1:BISNI, ASRS1:ASRS18, ASRS, PADUA1:PADUATotal,

ADHD.Y.N, GAD.Y.N, Soc.Anx.Y.N, OCD.Y.N, Gender, Age , Race.Binary, Education) %>%

filter(IAT.Total != "NA")

#transforming variables into factor variables

df3$ADHD.Y.N <- as.factor(df3$ADHD.Y.N); df3$GAD.Y.N <- as.factor(df3$GAD.Y.N); df3$Soc.Anx.Y.N <- as.factor(df3$Soc.Anx.Y.N)

df3$OCD.Y.N <- as.factor(df3$OCD.Y.N); ##df3$OCPD.Y.N <- as.factor(df3$OCPD.Y.N); df3$APD.Y.N <- as.factor(df3$APD.Y.N)

df3$Gender <- as.factor(df3$Gender) ; df3$Race.Binary <- as.factor(df3$Race.Binary); df3$Education <- as.factor(df3$Education)

df3$IAT.Class <- as.factor(df3$IAT.Class)

df4 <- df3; rm(df2)

# using the ID variable to split the IAT classes appropriately; during this process we confirm that the classification given by the team has been appropriate

ID <- 1:2503

df4$ID <- as.numeric(ID); df5 <- df4 %>% filter(ID < 1677); df5 = within(df5, (ID= ifelse(ID < 1662, "SA", "UK")))

df6 <- df4 %>% filter(ID > 1676); df6 = within(df6, (ID= ifelse(ID < 1677, "UK", "CHI")))

df4 <- rbind(df5, df6); df4$ID <- as.factor(df4$ID)

# now we have all three IAT classes but need to further split in PIU and non-PIU

df10A <- df4 %>% filter(IAT.Total < 50); df10A = within(df10A, (IAT50= ifelse(IAT.Total < 50, 0, 1)))

df10B <- df4 %>% filter(49 < IAT.Total) %>% filter(IAT.Total < 80);df10B = within(df10B, (IAT50= ifelse(IAT.Total < 80, 1, 2)))

df10C <- df4 %>% filter(IAT.Total > 79); df10C = within(df10C, (IAT50= ifelse(IAT.Total > 79, 1, 2)))

df11 <- rbind(df10A, df10B, df10C); df11 <- df11 %>% dplyr::select(-IAT.Class); df11$IAT50 <- as.factor(df11$IAT50)

#clearing up variables that are not used and filtering for missing values

df23 <- df11 %>% dplyr::select(BIS1:IAT50,ID, IAT.Total,

-ASRS7,-ASRS8,-ASRS9,-ASRS10,-ASRS11,-ASRS12,

-ASRS13,-ASRS14,-ASRS15,-ASRS16,-ASRS17,-ASRS18,-PADUATotal) %>%

filter(complete.cases(.))

df23 <- df23 %>% filter(Age>17) #filtering out noon-adults

df23 <- df23 %>% filter( Gender != 2) #filtering out transgender gender

df23table <- df23 # creating specific table for preparation of table 1 statistics

df23 <- df23 %>% mutate_each_(funs(scale), vars = c("Age")) # standardizing age

...

More code to create validation sets

...

# here begins the r code for prediction models

#first step creating metrics matrices to be populated with results in each replication

acc.mat <- matrix(0, 0,6); colnames(acc.mat) <- c("results.mtry" , "results.Accuracy", "results.Kappa", "results.AccuracySD", "results.KappaSD", "Test_accuracy")

vI.mat <- matrix(0, 0, 2); colnames(vI.mat) <- c("Overall", "names")

res.mat <- matrix(0, 0, 6); colnames(res.mat) <- c("Sensitivity", "Specificity", "PPV", "NPV", "Kappa", "F Measure")

auc.mat <- matrix(0, 0, 1); colnames(auc.mat) <- c("AUC")

pr.auc.mat <- matrix(0, 0, 1); colnames(pr.auc.mat) <- c("PR-AUC")

auc.plot.matrix.comp <- matrix(0,0,2)

pr.plot.matrix.comp <- matrix(0,0,2)

#setting number of replications

k = 50

#setting random seed to allow replication of results

set.seed(23)

#here begins the loop

for (i in 1:k) {

inTrain <- createDataPartition(y = dfSAR$IAT50, #using the caret package to partition data

p = 0.75, list = FALSE)

training <- dfSAR[as.vector(inTrain), ]

testing <- dfSAR[-as.vector(inTrain), ]

# aucrf <- AUCRF(IAT50 ~. , data = training, k0 = 1, pdel = 0.1, ranking=c("MDA"))

# this is another package if you want to optimize AUC metrics

# training <- training %>% select(one_of(aucrf$Xopt), IAT50)

training = within(training, (IAT50= ifelse(IAT50 == "0", "A", "B"))); training$IAT50 <- as.factor(training$IAT50) #to overcome a bug

# testing <- testing %>% select(one_of(aucrf$Xopt), IAT50)

testing = within(testing, (IAT50= ifelse(IAT50 == "0", "A", "B")));testing$IAT50 <- as.factor(testing$IAT50) #to overcome a bug

ctrl <- trainControl(method = "cv", #to control cross-validation parameters and solve this as a two class classification problem

classProbs = TRUE,

summaryFunction = twoClassSummary)

modFit<- train(IAT50 ~. , data = as.data.frame(training),

metric = "ROC", #another possibility is RMSE to try and predict the actual IAT score

method = "glm", #that would indicate the model of logistic regression glm (family binomial)

trControl = ctrl)

#this block below will start populating the variable importance matrix

varImp.matrix <- data.frame(varImp(modFit)[1], stringsAsFactors = TRUE)

varImp.matrix$names <- rownames(varImp.matrix)

vI.mat <- rbind(vI.mat, varImp.matrix)

#here we use the modFit trained above to make probabilistic predictions on the testing set

rf.test.prob = predict(modFit, newdata = testing, type = "prob")[,2]; rf.test.prob <- as.vector(rf.test.prob)

rf.test.pred <- prediction(rf.test.prob, testing$IAT50)

#and we assess roc and pr characteristics

auc <- performance(rf.test.pred,"auc")

auc <- unlist(slot(auc, "y.values"))

auc.mat <- rbind(auc.mat, auc)

auc.perf <- performance(rf.test.pred,"tpr","fpr")

auc.x.values <- unlist(slot(auc.perf, "x.values")); auc.y.values <- unlist(slot(auc.perf, "y.values"));

auc.plot.mat <- cbind(auc.x.values, auc.y.values); auc.plot.matrix.comp <- rbind(auc.plot.matrix.comp, auc.plot.mat)

pr.perf <- performance(rf.test.pred,"f") # pr.perf <- performance(rf.test.pred, "prec","rec")

pr.x.values <- unlist(slot(pr.perf, "x.values")); pr.y.values <- unlist(slot(pr.perf, "y.values"));

pr.plot.mat <- cbind(pr.x.values, pr.y.values); pr.plot.matrix.comp <- rbind(pr.plot.matrix.comp, pr.plot.mat)

pr.plot.mat <- data.table(pr.plot.mat); pr.plot.mat <- pr.plot.mat %>% filter(pr.y.values > 0)

pr.auc <- auc(pr.plot.mat$pr.x.values, pr.plot.mat$pr.y.values)

pr.auc.mat <- rbind(pr.auc.mat, pr.auc)

more code to complete the analysis

...

#

# Appendix References

Bishop, Christopher. 2006. *Pattern Recognition and Machine Learning*. New York, USA: Springer.

Breiman, Leo. 2001a. “Statistical Modeling: The Two Cultures (with Comments and a Rejoinder by the Author).” *Statistical Science* 16 (3): 199–231. doi:10.1214/ss/1009213726.

———. 2001b. “Random Forests.” *Machine Learning* 45 (1): 5–32. doi:10.1023/A:1010933404324.

Bureau, Alexandre, Josée Dupuis, Kathleen Falls, Kathryn L. Lunetta, Brooke Hayward, Tim P. Keith, and Paul Van Eerdewegh. 2005. “Identifying SNPs Predictive of Phenotype Using Random Forests.” *Genetic Epidemiology* 28 (2): 171–82. doi:10.1002/gepi.20041.

Cristianini, Nello, and John Shawe-Taylor. 2000. *An Introduction to Support Vector Machines and Other Kernel-Based Learning Methods*. Cambridge University Press.

Elsabbagh, Mayada, Agnes Volein, Gergely Csibra, Karla Holmboe, Holly Garwood, Leslie Tucker, Sanya Krljes, et al. 2009. “Neural Correlates of Eye Gaze Processing in the Infant Broader Autism Phenotype.” *Biological Psychiatry* 65 (1): 31–38. doi:10.1016/j.biopsych.2008.09.034.

Iwabuchi, Sarina, Peter F. Liddle, and Lena Palaniyappan. 2013. “Clinical Utility of Machine-Learning Approaches in Schizophrenia: Improving Diagnostic Confidence for Translational Neuroimaging.” *Neuropsychiatric Imaging and Stimulation* 4: 95. doi:10.3389/fpsyt.2013.00095.

Khodayari-Rostamabad, Ahmad, Gary M. Hasey, Duncan J. MacCrimmon, James P. Reilly, and Hubert de Bruin. 2010. “A Pilot Study to Determine Whether Machine Learning Methodologies Using Pre-Treatment Electroencephalography Can Predict the Symptomatic Response to Clozapine Therapy.” *Clinical Neurophysiology* 121 (12): 1998–2006. doi:10.1016/j.clinph.2010.05.009.

Nouretdinov, Ilia, Sergi G. Costafreda, Alexander Gammerman, Alexey Chervonenkis, Vladimir Vovk, Vladimir Vapnik, and Cynthia H. Y. Fu. 2011. “Machine Learning Classification with Confidence: Application of Transductive Conformal Predictors to MRI-Based Diagnostic and Prognostic Markers in Depression.” *NeuroImage*, Multivariate Decoding and Brain Reading, 56 (2): 809–13. doi:10.1016/j.neuroimage.2010.05.023.

Orrù, Graziella, William Pettersson-Yeo, Andre F. Marquand, Giuseppe Sartori, and Andrea Mechelli. 2012. “Using Support Vector Machine to Identify Imaging Biomarkers of Neurological and Psychiatric Disease: A Critical Review.” *Neuroscience and Biobehavioral Reviews* 36 (4): 1140–52. doi:10.1016/j.neubiorev.2012.01.004.

Patel, Meenal J., Carmen Andreescu, Julie C. Price, Kathryn L. Edelman, Charles F. Reynolds, and Howard J. Aizenstein. 2015. “Machine Learning Approaches for Integrating Clinical and Imaging Features in Late-Life Depression Classification and Response Prediction.” *International Journal of Geriatric Psychiatry* 30 (10): 1056–67. doi:10.1002/gps.4262.

Robnik-Sikonja, Marko. 2004. “Improving Random Forests.” In *Machine Learning, ECML 2004 Proceedings*. Berlin: Springer.

Rose, Sherri. 2013. “Mortality Risk Score Prediction in an Elderly Population Using Machine Learning.” *American Journal of Epidemiology* 177 (5): 443–52. doi:10.1093/aje/kws241.

Shrivastava, Amresh, Megan Johnston, Nilesh Shah, Larry Stitt, Shivanshu Shrivastava, and Avinash De Sousa. 2014. “Clozapine Response and Pre-Treatment EEG-Is There Some Kind of Relationship.” *Industrial Psychiatry Journal* 23 (1): 18–22. doi:10.4103/0972-6748.144951.

Stahl, Daniel, Andrew Pickles, Mayada Elsabbagh, Mark H. Johnson, and BASIS Team. 2012. “Novel Machine Learning Methods for ERP Analysis: A Validation from Research on Infants at Risk for Autism.” *Developmental Neuropsychology* 37 (3): 274–98. doi:10.1080/87565641.2011.650808.

Stone, M. 1974. “Cross-Validatory Choice and Assessment of Statistical Predictions.” *Journal of the Royal Statistical Society. Series B (Methodological)* 36 (2): 111–47. doi:10.2307/2984809.

Strobl, Carolin, James Malley, and Gerhard Tutz. 2009. “An Introduction to Recursive Partitioning: Rationale, Application, and Characteristics of Classification and Regression Trees, Bagging, and Random Forests.” *Psychological Methods* 14 (4): 323–48. doi:10.1037/a0016973.

Wall, D. P., J. Kosmicki, T. F. DeLuca, E. Harstad, and V. A. Fusaro. 2012. “Use of Machine Learning to Shorten Observation-Based Screening and Diagnosis of Autism.” *Translational Psychiatry* 2 (4): e100. doi:10.1038/tp.2012.10.
